# Inter- and Intra-Patient Repeatability of Radiomic Features from Multiparametric Whole-Body MRI in Patients with Metastatic Prostate Cancer

**DOI:** 10.3390/cancers16091647

**Published:** 2024-04-25

**Authors:** Ricardo Donners, Antonio Candito, Mihaela Rata, Adam Sharp, Christina Messiou, Dow-Mu Koh, Nina Tunariu, Matthew D. Blackledge

**Affiliations:** 1 University Hospital Basel, Petersgraben 4, 4031 Basel, Switzerland; 2 The Institute of Cancer Research, 15 Cotswold Road, Sutton SM2 5NG, UK; antonio.candito@icr.ac.uk (A.C.); mihaela.rata@icr.ac.uk (M.R.); adam.sharp@icr.ac.uk (A.S.); christina.messiou@icr.ac.uk (C.M.); dow-mu.koh@icr.ac.uk (D.-M.K.); nina.tunariu@icr.ac.uk (N.T.)

**Keywords:** radiomics, diffusion magnetic resonance imaging, neoplasm metastases

## Abstract

**Simple Summary:**

Prostate cancer bone metastases are a heterogonous disease with heterogeneous therapy-response, not adequately captured by one-dimensional imaging biomarker measurements. DWI and Dixon MRI radiomics analysis may tackle this shortcoming, but technical assessment of repeatability is an essential prerequisite before implementation. In this manuscript we identified whole-body MRI radiomics features in prostate cancer bone disease with good inter- and intra-patient repeatability. These features may be further explored to improve outcome predictions and therapy response assessment in prostate cancer patients.

**Abstract:**

(1) Background: We assessed the test–re-test repeatability of radiomics in metastatic castration-resistant prostate cancer (mCPRC) bone disease on whole-body diffusion-weighted (DWI) and T1-weighted Dixon MRI. (2) Methods: In 10 mCRPC patients, 1.5 T MRI, including DWI and T1-weighted gradient-echo Dixon sequences, was performed twice on the same day. Apparent diffusion coefficient (ADC) and relative fat-fraction-percentage (rFF%) maps were calculated. Per study, up to 10 target bone metastases were manually delineated on DWI and Dixon images. All 106 radiomic features included in the Pyradiomics toolbox were derived for each target volume from the ADC and rFF% maps. To account for inter- and intra-patient measurement repeatability, the log-transformed individual target measurements were fitted to a hierarchical model, represented as a Bayesian network. Repeatability measurements, including the intraclass correlation coefficient (ICC), were derived. Feature ICCs were compared with mean ADC and rFF ICCs. (3) Results: A total of 65 DWI and 47 rFF% targets were analysed. There was no significant bias for any features. Pairwise correlation revealed fifteen ADC and fourteen rFF% feature sub-groups, without specific patterns between feature classes. The median intra-patient ICC was generally higher than the inter-patient ICC. Features that describe extremes in voxel values (minimum, maximum, range, skewness, and kurtosis) showed generally lower ICCs. Several mostly shape-based texture features were identified, which showed high inter- and intra-patient ICCs when compared with the mean ADC or mean rFF%, respectively. (4) Conclusions: Pyradiomics texture features of mCRPC bone metastases varied greatly in inter- and intra-patient repeatability. Several features demonstrated good repeatability, allowing for further exploration as diagnostic parameters in mCRPC bone disease.

## 1. Introduction

Metastatic castration-resistant prostate cancer (mCRPC) is a lethal disease. Bone metastases develop in 90% of mCRPC patients and are a major cause of morbidity and mortality [1]. However, conventional anatomic imaging techniques, including CT, bone scans, and standard MRI are inadequate for the response assessment of malignant bone disease [2,3].

By contrast, whole-body MRI (WB-MRI), including diffusion-weighted imaging (DWI) and T1-weighted fat/water (Dixon) sequences, can assess the treatment response of bone disease [4]. DWI informs on tumour cellularity, whilst Dixon acquisition assesses the relative tissue fat content. Both techniques facilitate the identification, staging, and response assessment of bone metastases and may provide quantitative response biomarkers [4,5,6,7,8,9,10,11]. The DWI-derived apparent diffusion coefficient (ADC) and Dixon MRI-derived relative fat-fraction percentage (rFF%) correlate with tumour cellularity in prostate cancer bone metastases and show increases with therapy response [5,6,12,13,14,15,16,17,18]. However, the simple averaging of imaging biomarker values within delineated regions of interest (ROIs) fails to capture the commonly heterogeneous appearance of mCRPC disease. Studies have suggested that more complex evaluations of tumour texture features can improve therapy outcome predictions [19,20,21].

The computerised extraction of quantitative features from medical images to describe different cancer phenotypes is called “radiomics”. Many radiomic features have been described, but there is still no routine implementation in clinical practise for mCRPC. Several factors contribute to this disparity between research and clinical application, which include the lack of an integral clinical pipeline for data curation, a lack of capacity for tumour annotation, and no clinical processing tools for the disease. It is not established which features provide consistent, repeatable results, which can be harnessed in a test–retest setting, to inform on relevant changes between baseline and follow-up imaging in cancer patients undergoing therapy [22]. Knowing a feature’s repeatability is important—if a parameter shows poor repeatability, its predictive power is low; thus, excellent repeatability can be considered a prerequisite for meaningful parameter selection among the large number of radiomic features [23]. The study of MRI radiomics repeatability is challenging, because in contrast to CT, there is no inherent normalisation of signal intensities, making test–re-test comparisons between examinations difficult. DWI-derived ADC and Dixon-derived rFF% maps may tackle this shortcoming, as both parametric maps offer inherent normalisation, enabling inter-study comparisons [24].

To date, no radiomics repeatability study has been published in conjunction with the MRI assessment of metastatic bone disease. Without technical validation, no meaningful radiomics features can be identified, as was highlighted by expert consensus statements [25,26]. Consequently, the purpose of this study is to contribute to the work on this knowledge gap by assessing the test–re-test repeatability of radiomic features in mCPRC bone disease assessed on WB-MRI DWI and T1-weighted Dixon sequences. We considered all texture features included in the open-source Pyradiomics package, which are implemented according to consensus definitions of the Imaging Biomarker Standardisation Initiative (IBSI) [26,27].

## 2. Materials and Methods

### 2.1. Study Design

This prospective repeatability study was approved by the local research and ethics committee. Prostate cancer patients were recruited and consented in one institution. The study inclusion criteria were the histopathology diagnosis of prostate cancer, history of bone metastases, castration-resistant disease, and no contraindication for MRI acquisition. The exclusion criterion was contraindications for MRI acquisition. In total, eleven mCRPC patients were recruited.

### 2.2. Imaging Acquisition

Initial and repeat WB-MRI acquired on a Siemens MAGNETOM Aera 1.5T MRI system (Siemens Healthineers, Erlangen, Germany) were evaluated. Patients were scanned twice in one setting, with repositioning between the examinations. The median time interval between the initial and re-test imaging sequences was 54 min. The imaging protocol included DWI and CAIPIRINHA (Controlled Aliasing in Parallel Imaging Results in Higher Acceleration)-accelerated T1-weighted Dixon MRI, as shown in Table 1. ADC and rFF% maps were calculated using in-house routines.

### 2.3. Disease Delineation

**Figure 1:**
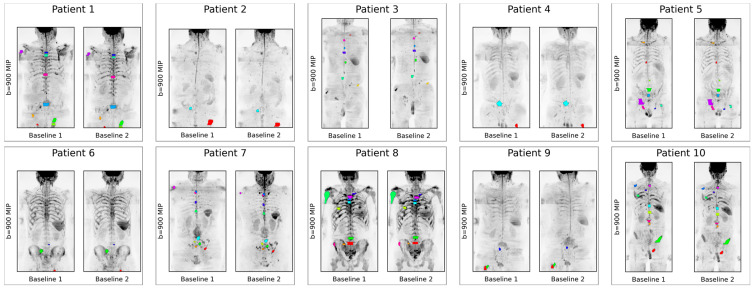
Maximum intensity projections (MIPs) of the high-b-value images for all 10 repeatability patients available to the study, including regions of interest displayed as colour overlays (each individual lesion displayed as a different colour).

Disease delineation was performed on commercially available post-processing software (OsiriX, version 56, PixmeoSARL Bernex, Switzerland) by a dedicated radiology fellow with four years of experience in the functional imaging of malignant bone disease. In each patient, up to 10 bone metastases were chosen as target lesions, facilitating the identification of inter-lesion heterogeneity. Lesions were selected across the body (where present) in the cervical spine, thoracic spine, lumbar spine, sacrum, pelvis, ribs, shoulders, and long bones. Target bone metastases were defined as a focal lesion with a low signal on rFF% images (<20%) compared with adjacent bone marrow, together with an unsuppressed high signal on b50 and b900 DWI and a mean ADC value of <1400 × 10^−^^6^ mm^2^/s. Lesions with mean ADC > 1400 × 10^−^^6^ mm^2^/s were evaluated together with previous imaging and were suitable for inclusion when they showed unequivocal increases or decreases in size and/or in ADC ≥ 30% (4). We did not analyse lesions < 1 mL in volume. (Figure 1).

The whole target lesion was segmented in consecutive slices in the b900 and rFF% images. The b900 segmentation masks were copied onto the corresponding ADC maps. As there was no ground truth, the absolute accuracy of segmentation was not evaluated as part of the study.

### 2.4. Extraction of Radiomics Features

Radiomics features were derived using the Pyradiomics toolbox [27], including the first order, shape, grey-level co-occurrence matrix (GLCM), grey-level run length matrix (GLRLM), grey-level size zone matrix (GLSZM), grey-level dependence matrix (GLDM), and neighbouring grey tone difference matrix (NGTDM) features (total of 106 features). Feature extraction definition files are presented in the Supplementary Information S1 and S2. As many derived features demonstrate heteroscedastic repeatability, all features were subsequently log-transformed, except for skewness (first order), minimum (first order), correlation (GLCM), and cluster shade (GLCM), which were observed to have both positive and negative and/or zero values [28]. Furthermore, the inverse difference normalised (GLCM) and inverse difference moment normalised (GLCM) were transformed according to y = 1−x, and informational measure of correlation (GLCM) was normalised by y = 1−x prior to log-transformation. No wavelet-filtered features were investigated. We used a fixed bin size of 100 × 10^−6^ mm^2^/s and 3.333% for ADC and rFF% maps, respectively, such that approximately 30 bins were applied in each case.

### 2.5. Repeatability Model

**Figure 2:**
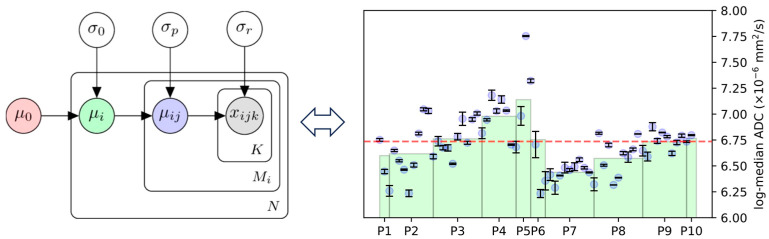
A Bayesian network of per-lesion measurements, x, within a whole-body MRI experiment. The model consists of three hierarchical normal distributions for the population, $\left(\mu_{0},\sigma_{0}\right)$, the ith patient $\left(\mu_{i},\sigma_{p}\right)$, and the jth lesion within the patient $\left(\mu_{ij},\sigma_{r}\right)$. Note that whilst separate mean values μ are determined for each patient/lesion, global values for standard deviation σ are defined.

We considered the (log-transformed) measurements derived from each lesion within each patient to be derived from a hierarchical model, graphically represented as a Bayesian network in Figure 2.

We denote the kth repeat measurement made in the jth lesion of patient i as $x_{ijk}$, where $k\in \left\{1,2\right\}$, $j\in \left\{1,\ldots ,M_{i}\right\}$, and $i\in \left\{1,\ldots ,N\right\}$. Each measurement is assumed to be normally distributed about the true lesion value, $\mu_{ij}$, with inter-measurement error, $\sigma_{r}$, each lesion value being normally distributed about the true patient value $\mu_{i}$ with intra-patient variation, $\sigma_{p}$, and each patient value being normally distributed about the population average $\mu_{0}$ with inter-patient variation, $\sigma_{0}$: $x_{ijk}∼N(\mu_{ij},\sigma_{r})$, $\mu_{ij}∼N(\mu_{i},\sigma_{p})$, and $\mu_{i}∼N(\mu_{0},\sigma_{0})$. A key advantage to this model is that it allows us to disentangle variation amongst lesions within an individual patient from variation between different patients and thus understand whether a particular measurement is sensitive to changes occurring on a per-lesion and/or per-patient level. Once estimates of the parameters from this model were determined, we extracted several summary statistics, in line with those from the traditional repeatability literature.
**Equation****Description**$RC={1.96\sqrt{2}\sigma }_{r}$Repeatability coefficient. Useful in the context of assessing response after treatment. Any change above +RC or below −RC is considered to be statistically significant and thus might be a direct result of treatment rather than due to measurement error (assuming a p-value of 0.05).${ICC}_{\delta }=\frac{\sigma_{p}^{2}}{\sigma_{p}^{2}+\sigma_{r}^{2}}$ $\left(0\leq {ICC}_{\delta }\leq 1\right)$Intra-patient intraclass correlation. Compares the magnitude of the inter-measurement error with intra-patient variation in lesion values. A value closer to 1 indicates better measurement repeatability in the context of measuring changes to individual lesions.${ICC}_{\Delta }=\frac{\sigma_{0}^{2}}{\sigma_{0}^{2}+\sigma_{r}^{2}}$ $\left(0\leq {ICC}_{\Delta }\leq 1\right)$Inter-patient intraclass correlation. Compares the magnitude of the inter-measurement error with inter-patient variation in lesion values. A value closer to 1 indicates better measurement repeatability in the context of measuring changes with groups of lesions within each patient.$wlCV=\sqrt{\frac{1}{\sum_{i}M_{i}}\sum_{i,j}\frac{\sigma_{r}^{2}}{\mu_{ij}^{2}}}$ (wlCV≥0)Average within-lesion coefficient of variation. Describes the magnitude of inter-measurement error in the context of true lesion values. A large value represents potentially poor repeatability compared with the expected values for each lesion.$blCV=\sqrt{\frac{1}{N}\sum_{i}\frac{\sigma_{p}^{2}}{\mu_{i}^{2}}}$ (blCV≥0)Average between-lesion coefficient of variation. Describes the magnitude of inter-lesion variation in the context of average patient values. A large value represents higher intra-patient heterogeneity.$bCV=\frac{\sigma_{0}}{\mu_{0}}$ (bCV≥0)Between-patient coefficient of variation. Describes the magnitude of inter-patient variation in the context of the average population value. A large value represents higher inter-patient heterogeneity.$LoA=\left(e^{\pm 1.96\sqrt{2}\sigma_{r}^{\prime }}-1\right)$ ×100%Limits of agreement (log-transformed features only). Defines the percentage difference after treatment needed to deem that change significantly different.

It is important to note that in this setting, we defined $\sigma_{p}$ as a population-wide parameter, where, in theory, it might be estimated for each patient. However, given the numbers of lesions we encounter in certain patients, it can become very difficult to meaningfully estimate this parameter on a per-patient basis. Furthermore, by considering it as a population-wide estimate, it is much simpler to compare it with the measurement error $\sigma_{r}$.

We also defined a bias parameter ε in the model that represents the average difference between both baseline measurements: $x_{ij1}∼N(\mu_{ij},\sigma_{r})$ and $x_{ij2}∼N(\mu_{ij}+\varepsilon ,\sigma_{r})$. This allowed us to confirm that no systematic bias occurred between both baseline measurements. The total number of parameters in this model was $8+N+\sum_{i}M_{i}$.

### 2.6. Model Fitting

We used the Markov Chain Monte Carlo (MCMC) optimisation with Stan for hierarchical modelling [29]. This technique draws samples from the posterior probability distribution of model parameters given the available data, thereby fully characterising uncertainty in parameter estimation (and subsequently generated statistics). The Stan code for our implementation is presented in Supplementary S3.

Before sampling, we standardised our data using the convention $x_{ij1}^{\prime }=\left(x_{ij1}-\bar{x_{1}}\right)/\sqrt{Var\left(x_{1}\right)}$ and $x_{ij2}^{\prime }=\left(x_{ij2}-\bar{x_{1}}\right)/\sqrt{Var\left(x_{1}\right)}$, where $\bar{x_{1}}$ and $Var\left(x_{1}\right)$ represent the mean and variance in the data from baseline measurement 1, respectively. Furthermore, we initialise the parameter values for sampling using the following:
${\hat{\mu }}_{ij}^{0}=\frac{1}{2}\left(x_{ij1}+x_{ij2}\right)$${\hat{\sigma }}_{r}^{0}=\sqrt{\frac{1}{2\sum_{i}M_{i}}\sum_{i,j}{\left(x_{ij1}-x_{ij2}\right)}^{2}}$${\hat{\mu }}_{i}^{0}=\frac{1}{M_{i}}\sum_{j}{\hat{\mu }}_{ij}^{0}$${\hat{\sigma }}_{p}^{0}=\sqrt{\frac{1}{\sum_{i}M_{i}-N}\sum_{i,j}{\left({\hat{\mu }}_{ij}^{0}-{\hat{\mu }}_{i}^{0}\right)}^{2}}$${\hat{\mu }}_{0}^{0}=\frac{1}{N}\sum_{i}{\hat{\mu }}_{i}^{0}$${\hat{\sigma }}_{0}^{0}=\sqrt{\frac{1}{N-1}\sum_{i}{\left({\hat{\mu }}_{i}^{0}-{\hat{\mu }}_{0}^{0}\right)}^{2}}$${\hat{\varepsilon }}^{0}=\frac{1}{\sum_{i}M_{i}}\sum_{i,j}\left(x_{ij2}-x_{ij1}\right)$


Prior distributions for parameters $\sigma_{0}$, $\sigma_{p}$, and $\sigma_{r}$ were set to be half-Cauchy distributions with location 0 and scale 5, whilst priors for $\mu_{0}$ and ε were zero-mean normal distributions with a standard deviation of 10. Checks were made that the range of these distributions covered the range of initial model parameters ${\hat{\sigma }}_{r}^{0}$, ${\hat{\sigma }}_{p}^{0}$, ${\hat{\sigma }}_{0}^{0}$, ${\hat{\varepsilon }}^{0}$, and ${\hat{\mu }}_{r}^{0}$ for all radiomic features investigated in this study. Sampling parameters included the following: number of chains = 3, number of samples = 2000, number of warmup samples = 500, no thinning, and fixed random seed initialisation.

To assess the independence of successive samples and good mixing of multiple sampling chains, we used the Gelman–Rubin R-hat ($\hat{R}$) convergence diagnostic: Calculated as the ratio of the pooled variance of parameters across multiple chains to the average variance within each individual chain, good mixing was observed as $\hat{R}\to 1$. Our sampling regime consisted of checking that 99% of all parameters had $\hat{R}\leq 1.02$; otherwise, samples were rejected and repeated up to 10 times. In our study, this schema needed, at most, two retries until adequate convergence was found over all radiomics features considered.

Fixed thresholds for repeatability interpretation can be problematic [30]. Consequently, we compared the ICCs of the extracted texture features to the mean ADC and mean rFF% ICCs to allow for some classification. Features with equal or greater ICCs than these reference metrics were considered to offer good repeatability.

## 3. Results

As one patient did not show bone metastases on WB-MRI, a total of 10 mCRPC patients with a median age of 67.5 years were included for analysis. All patients had undergone all lines of standard-of-care treatment and were undergoing systemic therapy at the time of study inclusion. In one patient, Dixon imaging had been performed with erroneous pixel spacing during one of the repeat measurements, and so this patient was removed from rFF% analysis. In total, 65 delineated target lesions were used for ADC analysis and 47 for rFF% analysis.

Median repeatability model parameters for all radiomics features are presented in Supplementary S4 and S5. For all features, for both ADC and rFF%, there was no evidence of significant bias.

**Figure 3:**
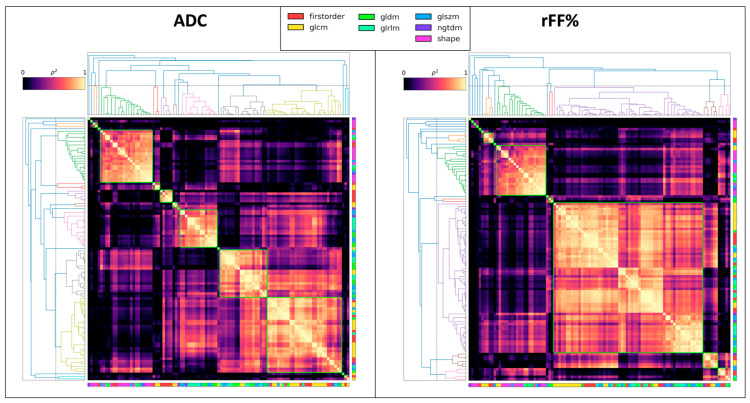
Correlation heatmaps of radiomics features for both ADC and fat fraction values. It is noted that a large proportion of the radiomics features are highly correlated; 15 sub-groups are identified as ADC-derived features, whilst 14 are identified for fat-fraction-derived features, using hierarchical clustering with a pairwise Spearman correlation threshold of 1 − $\rho^{2}$ = 0.51. A high-resolution copy of this figure is provided in Supplementary Information S6, which depicts the name of all features.

Heatmaps of pairwise distance correlation for radiomic features are presented in Figure 3 (and Supplementary S6). Fifteen and fourteen sub-groups were identified for ADC-derived and rFF%-derived features, respectively. No specific patterns were identified in terms of the radiomic feature classes that were identified within these groups, other than typical parameters such as “10th percentile”, “90th percentile, “median”, “mean”, and “root-mean-squared” being grouped together in both cases. Although the first-order “minimum” was identified to exist in its own subgroup for rFF%, we note that this feature was zero for many lesions and thus likely not a reliable biomarker.

**Figure 4:**
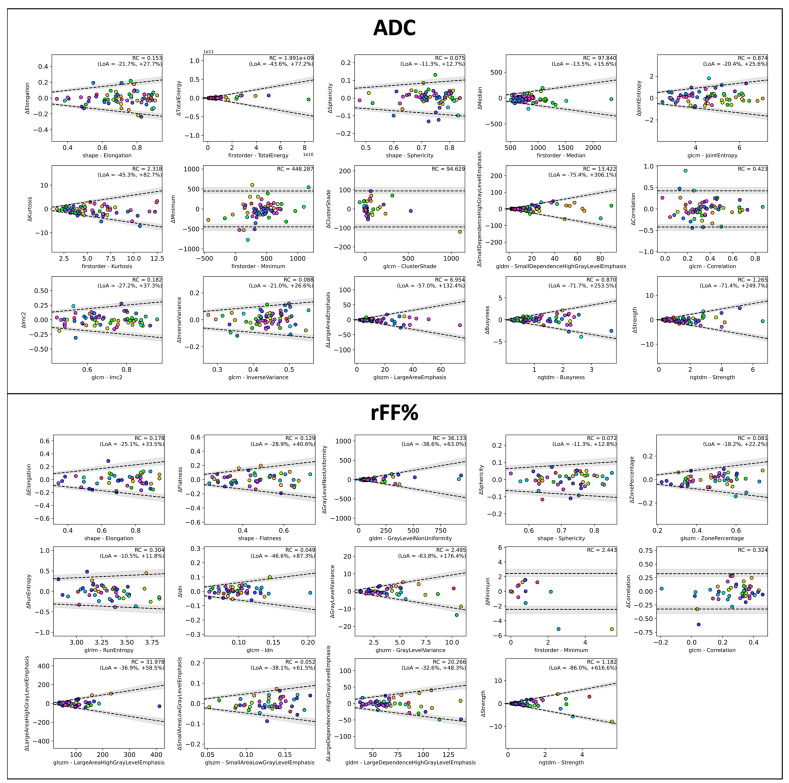
Bland–Altman plots for radiomics features from our study. Presented are those features that demonstrated the best median ${ICC}_{\delta }$ within each correlation group. The scatter plots are color-coded according to patient to demonstrate the inter-patient variability. Black dashed lines represent limits of repeatability (95% confidence interval illustrated as grey areas), and the values of the repeatability coefficient (RC) and limits of agreement (LoA) are provided.

Bland–Altman plots for a single representative radiomics feature from each correlated sub-group are presented in Figure 4, where in each sub-group, the feature with the maximum intra-patient ICC is presented. Repeatability limits are adjusted to account for the observed linearity using the method of Euser et al. [28].

**Figure 5:**
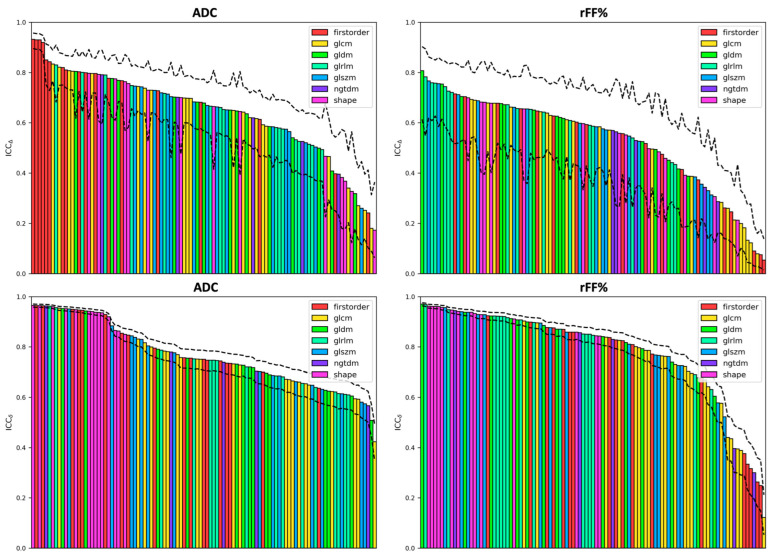
Waterfall plots of the inter- and intra-patient intraclass correlation coefficients for all radiomics features (${ICC}_{\Delta }$ top row, and ${ICC}_{\delta }$ bottom row, respectively). Values for ADC measurements are presented in the left column, whilst values for fat fraction measurements are shown in the right column. Bars are colour-coded according to the radiomic feature type, and dashed lines represent the interquartile range of ICC values. A high-resolution copy of this figure is provided in Supplementary Information S7, which depicts the name of all features.

Waterfall plots for the median intra- and inter-patient ICCs are presented in Figure 5 (and Supplementary S7) for all radiomics features investigated, along with inter-quartile ranges (IQRs). The median intra-patient ICC, ${ICC}_{\delta }$, was generally higher than the median inter-patient ICC, ${ICC}_{\Delta }$, though the uncertainty in the estimated ${ICC}_{\Delta }$ was also generally much larger (due to a smaller effective sample size).

**Figure 6:**
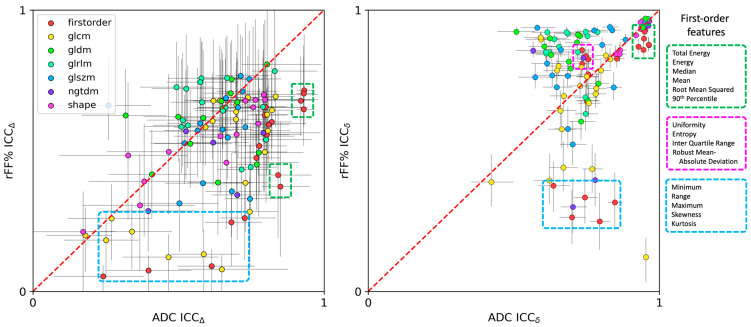
Scatter plots for inter- and intra-patient intraclass correlation coefficients for all radiomics features (${ICC}_{\Delta }$ left, and ${ICC}_{\delta }$ right, respectively), comparing metrics between ADC and fat fraction. The red dashed line represents the line of equality, and interquartile ranges for the measured ICC are displayed as error bars around each scatter point. Our data seem to indicate that the inter-lesion ICC for fat fraction is significantly lower than for ADC for features that capture the extremes in data and may not be robust to outliers (blue dashed box). However, as might be expected, features that capture data averages demonstrate high ${ICC}_{\delta }$ in both cases (green dashed box).

This is echoed in Figure 6, which presents scatter plots of ${ICC}_{\Delta }$ and ${ICC}_{\delta }$ for both ADC and rFF%. Features that describe extremes in voxel values (minimum, maximum, range, skewness, and kurtosis) appear to have much lower ${ICC}_{\delta }$ values for rFF% than for ADC. Conversely, many texture features appear to have higher ${ICC}_{\delta }$ values for rFF% than for ADC. It was difficult to interpret ${ICC}_{\Delta }$ differences between both quantitative metrics, as error bars were much larger, and therefore, any trends needed to be interpreted with caution.

### Comparison of Features with the Reference Metrics

The mean ADC and mean rFF% were chosen as reference metrics to allow for the assessment and comparison of radiomics features’ inter- and intra-patient repeatability. Supplementary S4 and S5 show the repeatability measurements for all analysed features.

The inter-patient ICC, ${ICC}_{\Delta }$, for the mean ADC was 0.93. Among the other 17 first-order features only the median and root-mean-squared showed equivalent ${ICC}_{\Delta }$ values. None of the 22 glcm, 14 gldm, 16 glrlm, 16 glszm, 5 ngtdm, or 14 shape features showed comparable ${ICC}_{\Delta }$ values (group maximum 0.74–0.83). The mean ADC intra-patient ICC, ${ICC}_{\delta }$, was 0.95. Three first-order, one glcm, one gldm, two glrlm, one glszm, one ngtdm, and five shape features yielded equivalent or higher ${ICC}_{\delta }$ values (0.95–0.97).

The mean rFF% inter-patient ICC, ${ICC}_{\Delta }$, was 0.70. The median rFF% ${ICC}_{\Delta }$ was 0.71. The gldm parameters GrayLevelNonUniformity and SmallDependenceEmphasis, 6/16 glrlm, 3/16 glszm, and 1/14 shape features showed equivalent or higher ${ICC}_{\Delta }$ values (group maximum 0.70–0.81). All features in the glcm and ngtdm groups had lower ${ICC}_{\Delta }$ values (maximum 0.69 and 0.57, respectively). However, Bayesian sampling of ${ICC}_{\Delta }$ revealed relatively large confidence intervals in parameter estimation for all features, and thus, overlap between feature precision was apparent in all groups. The mean rFF% intra-patient ${ICC}_{\delta }$ was 0.90. The median rFF% intra-patient ${ICC}_{\delta }$ was 0.92. Two glcm, six gldm, nine glrlm, five glszm, one ngtdm, and eleven shape features showed equivalent or higher ${ICC}_{\delta }$ values (group maximum 0.90–0.97).

## 4. Discussion

In this study of the intra- and interpatient repeatability of radiomic features in mCRPC bone disease, we found that the intra-patient ICC, ${ICC}_{\delta }$, was generally higher than inter-patient ICC, ${ICC}_{\Delta }$. This suggests that Pyradiomics features are more stable and thus might be more sensitive to changes occurring for individual lesions rather than total-body measurements.

Regarding ADC map radiomic analyses, the most repeatable features were shape-based or first-order features, demonstrating excellent repeatability (${ICC}_{\Delta }$ and ${ICC}_{\delta }$ > 0.8). We note that many first-order features are highly correlated (mean, median, and root-mean-squared, for example), as shown by the fact that only 15 uncorrelated sub-groups were found from our correlation analysis of ADC features. The mean ADC is a commonly used biomarker in cancer imaging, and part of contemporary imaging and interpretation guidelines for prostate cancer bone disease [4], and has been shown to correlate negatively with tumour cellularity [5,31]. It is considered to offer good measurement repeatability and is therefore commonly employed for malignant bone marrow lesion comparison in a test–re-test setting [6,32]. Nonetheless, we identified fourteen texture features with equivalent ${ICC}_{\delta }$ values, with at least one being from each feature class. These features likely infer information about the heterogeneity of tumour cellularity, which may be compared between imaging time points, allowing for the monitoring of cancer evolution in patients undergoing oncology therapy.

Among texture classes for rFF% repeatability, the best performance was found for grey-level non-uniformity (GLDM and GLRLM), though only the GLDM version demonstrated ${ICC}_{\Delta }>0.8$. Multiple texture features from various feature groups demonstrated equivalent or higher ICCs when compared to the mean rFF% (${ICC}_{\Delta }$ ≥ 0.7 and ${ICC}_{\delta }$ > 0.9). Similar to the ADC, rFF% can also provide information on bone metastases and their evolution under cancer therapy—while a vital metastasis is assumed to contain no fat, a return of fatty bone marrow may suggest favourable response to therapy. The latter may be detected by the comparison of rFF% features between baseline and follow-up MRI.

When comparing the repeatability of radiomics texture features, GLRLM, GLDM, and GLSZM features generally had higher ${ICC}_{\delta }$ values for rFF% maps than for equivalent features derived from ADC maps. Many first-order feature ${ICC}_{\delta }$ values were equivalent between rFF% and ADC, with the exception of those that describe extremes in the voxel values including minimum, maximum, range, skewness, and kurtosis, which had significantly lower ${ICC}_{\delta }$ values than those computed using ADC. Shape-based features demonstrated similar ICC values between ADC and rFF%, which is likely because they should be independent of the imaging modality from which they were derived.

Recently, researchers applied radiomics to detect visually non-perceivable prostate cancer bone metastases on CT [33]. Hounsfield units are inherently normalised, which facilitates inter-study comparisons. By contrast, MRI signal intensity values are relative; however, ADC and rFF% maps provide inherent normalisation, enabling inter-study comparison.

In primary cancer of the prostate gland, research into MRI-derived radiomics is ahead of the current body of literature on radiomics in bone metastases. Two recent studies identified 12 and 15 features, respectively, extracted from pre-treatment T2- and dynamic contrast-enhanced T1-weighted MRI of the gland, which were significantly associated with the presence of bone metastases [34,35]. One may hypothesise that mCRPC bone metastases’ radiomics may likewise be used as predictors of response or even overall patient survival in future scenarios. Published research does not yet provide evidence for this hypothesis. Nonetheless, researchers have found that DWI-derived radiomics may be used to classify spinal tumours [36], dynamic contrast-enhanced spine MRI-derived radiomics can discriminate between lung cancer and non-lung cancer spine metastases [37], and MRI radiomics may be able to differentiate between malignant and benign spinal lesions [38], with more evidence to be expected in the near future.

Our study results suggest that several Pyradiomics features derived from ADC and rFF% maps have sufficiently high levels of repeatability to be utilised for predictive, diagnostic models. We identified ADC and rFF% radiomics with good repeatability in a test–re-test setting, which may contribute to a better understanding of the heterogenous responses seen in metastatic bone disease in mCRPC based on DWI/rFF alone. Our results are unique to this dataset. Consequently, general recommendations on which features may yield the highest diagnostic value in a follow-up setting in a patient undergoing oncology treatment cannot be made. For the common scenario of mCRPC patients undergoing repeat examinations for the surveillance of malignant bone disease, we have identified several parameters with a sufficient level of repeatability to be tested in future studies. Moreover, repeatability studies on conventional, single imaging biomarkers usually aim to determine limits of agreement to allow for the identification of meaningful parameter change thresholds in a test–re-test scenario—“meaningful change” is the measured difference in the quantitative imaging biomarker between two time points, which represents a true biological effect, such as response to therapy, rather than measurement variability. A quantitative parameter change between two time points which is larger than the determined LoA or repeatability coefficient can be considered meaningful. In this study, we clearly demonstrated linear agreement between the tested radiomics features. However, with the current level of evidence, we do not consider it sensible to conclude fixed parameter thresholds for the tested features.

This study has several limitations. First, only 10 patients were included. For the requirement of repeat scans, repeatability studies are time and labour intensive and usually include few patients. This is a key motivator for the development of our novel Bayesian pipeline for analysing repeatability data in whole-body MRI studies. Second, the diagnostic performance of the analysed texture features was not evaluated, as this is beyond the scope of the manuscript. Third, multiple factors affect feature repeatability, including the consistency of lesion segmentation between test and re-test examination. Although an experienced radiologist performed these measurements, a degree of variation must be expected. This is representative of clinical practise, where lesion measurements and comparisons will not be perfectly matched. A baseline ex vivo phantom study was beyond the scope of this article, but previous authors noted the good test–retest repeatability of DWI and ADC radiomics, acknowledging the limited implications for in vivo analyses [39].

Finally, the use of ICCs to compare repeatability across different biomarkers can be problematic [40]. For any response biomarker to be effective, two conditions should be met: Firstly, it must be precise enough to reliably detect genuine changes in the tumour property of interest, meaning the repeatability coefficient should be considerably smaller than the change anticipated after treatment. Secondly, the biomarker should exhibit a significant change in response to successful treatment, implying that the expected effect size is substantially greater than the measurement error, as determined using a repeatability assessment. While an ICC does not directly measure these conditions, it can still offer some insights into the relative repeatability of different biomarkers against their expected range of variation within a given patient cohort. However, without post-treatment data, an ICC can only suggest, not confirm, goodness of repeatability, and interpretations based on an ICC should be approached with caution. In determining the clinical utility of a biomarker, it is advisable to consider additional indices of repeatability, beyond the ICC (including the repeatability coefficient and coefficient of variation, also presented in this article), that better capture the precise nature required for clinical application.

## 5. Conclusions

In conclusion, mCRPC bone metastases Pyradiomics texture features vary greatly in inter- and intra-patient repeatability. In the presented dataset, we were not able to determine several universally stable features; however, we found several features with good repeatability, allowing for their further exploration as diagnostic parameters in mCRPC bone disease.

## Figures and Tables

**Table 1:** MRI acquisition parameters, DWI—diffusion-weighted imaging, GE—gradient-echo.

Parameter	DWI	rFF
b-values	B50, b600 b900	
TE	69	2.39
TR	11,300	7.63
Slice	6 mm	5 mm
Inversion	STIR 180	
Averages	3-5-5	1
Slice spacing	6	6
Px bandwidth	1955	400
Aqu Matrix	128 × 104	256 × 156
Image matrix	256 × 208	256 × 208
Flip angle	90	10

## Data Availability

Data are not publicly available but can be obtained from the corresponding author upon reasonable request.

## Supplementary Materials

The following supporting information can be downloaded at: https://www.mdpi.com/article/10.3390/cancers16091647/s1, S1–S4: Tables of ADC repeatability statistics for each radiomic features tested. Median values are given with 95% confidence interval provided in brackets (other than for R-hat statistics, where the minimum and maximum over all parameters is demonstrated); S5: Tables of Fat Fraction repeatability statistics for each radiomic features tested. Median values are given with 95% confidence interval provided in brackets (other than for R-hat statistics, where the minimum and maximum over all parameters is demonstrated); S6: High resolution version of Figure 3 that includes features names; S7: High resolution version of Figure 5 that includes features names.
