# Supplementary material for: Inter- and Intra-Patient Repeatability of Radiomic Features from Multiparametric Whole-Body MRI in Patients with Metastatic Prostate Cancer
Source: Cancers (Basel). 2024 Apr 25;16(9):1647. doi: 10.3390/cancers16091647 (PMC11083580; doi:10.3390/cancers16091647)
Supplement: Supplementary file 1 [file cancers-16-01647-s001.zip › Supplementary S1-S3.pdf]

**Supplementary S1**

Pyradiomics definitions file for ADC radiomic features calculations.


```yaml
imageType:
  Original: {}

featureClass:
  firstorder:
  shape:
  glcm:
    - 'Autocorrelation'
    - 'JointAverage'
    - 'ClusterProminence'
    - 'ClusterShade'
    - 'ClusterTendency'
    - 'Contrast'
    - 'Correlation'
    - 'DifferenceAverage'
    - 'DifferenceEntropy'
    - 'DifferenceVariance'
    - 'JointEnergy'
    - 'JointEntropy'
    - 'Imc1'
    - 'Imc2'
    - 'Idm'
    - 'Idmn'
    - 'Id'
    - 'Idn'
    - 'InverseVariance'
    - 'MaximumProbability'
    - 'SumEntropy'
    - 'SumSquares'
  glrlm:
  glszm:
  gldm:
  ngtdm:

setting:
  normalize: false
  interpolator: 'sitkLinear'
```

resampledPixelSpacing: [1.6796875, 1.6796875, 6.0]

padDistance: 10

preCrop: true

minimumROISize: 10

force2D: true

force2Ddimension: 2

binWidth: 100

voxelArrayShift: 0

symmetricalGLCM: true

**Supplementary S2**

Pyradiomics definitions file for fat fraction radiomic features calculations.


```
imageType:
  Original: {}

featureClass:
  firstorder:
  shape:
  glcm:
    - 'Autocorrelation'
    - 'JointAverage'
    - 'ClusterProminence'
    - 'ClusterShade'
    - 'ClusterTendency'
    - 'Contrast'
    - 'Correlation'
    - 'DifferenceAverage'
    - 'DifferenceEntropy'
    - 'DifferenceVariance'
    - 'JointEnergy'
    - 'JointEntropy'
    - 'Imc1'
    - 'Imc2'
    - 'Idm'
    - 'Idmn'
    - 'Id'
    - 'Idn'
    - 'InverseVariance'
    - 'MaximumProbability'
    - 'SumEntropy'
    - 'SumSquares'
  glrlm:
  glszm:
  gldm:
  ngtdm:

setting:
  normalize: false
  interpolator: 'sitkLinear'
  resampledPixelSpacing: [1.6796875, 1.6796875, 5.0]
  padDistance: 10
```

preCrop: true

minimumROISize: 10

force2D: true

force2Ddimension: 2

binWidth: 3.333

voxelArrayShift: 0

symmetricalGLCM: true

**Supplementary S3**

Below is the Stan script for drawing parameter samples given input data.

```
data {
    int<lower=0> N_tot; // Total number of samples (lesions)
    int<lower=0> N;     // Number of patients
    int<lower=0> M[N];  // Number of samples per patient (Note sum(M) == N)
    vector[N_tot] x1;   // Baseline measurements 1
    vector[N_tot] x2;   // Baseline measurements 2
}
parameters {
    real mu_0;              // Population mean
    real<lower=0> sigma_0;  // Inter-patient variation standard deviation
    vector[N] mu_i;         // Patient means
    real<lower=0> sigma_p;  // Intra-patient variation standard deviation
    vector[N_tot] mu_ij;    // Lesion means
    real<lower=0> sigma_r;  // Repeatability standard deviation
    real bias;              // Bias between double baseline measurements
}
model {
    // Priors
    mu_0 ~ normal(0, 10);
    bias ~ normal(0, 10);
    sigma_0 ~ cauchy(0, 5);
    sigma_p ~ cauchy(0, 5);
    sigma_r ~ cauchy(0, 5);

    // Likelihoods
    int pos = 1;
    for (n in 1:N) {
        mu_i[n] ~ normal(mu_0, sigma_0);
        for (m in pos:pos+M[n]-1) {
            mu_ij[m] ~ normal(mu_i[n], sigma_p);
            x1[m] ~ normal(mu_ij[m], sigma_r);
            x2[m] ~ normal(mu_ij[m] + bias, sigma_r);
        }
        pos = pos + M[n];
    }
}
```
