# Supplementary material for: Inter- and Intra-Patient Repeatability of Radiomic Features from Multiparametric Whole-Body MRI in Patients with Metastatic Prostate Cancer
Source: Cancers (Basel). 2024 Apr 25;16(9):1647. doi: 10.3390/cancers16091647 (PMC11083580; doi:10.3390/cancers16091647)
Supplement: Supplementary file 1 [file cancers-16-01647-s001.zip › Supplementary S4.pdf]

firstorder

Name	ICC inter (95% CI)	ICC intra (95% CI)	wlCV (95% CI)	blCV (95% CI)	bCV (95% CI)	RC (95% CI)	bias (95% CI)	-LoA (%) (95% CI)	+LoA (%) (95% CI)	R-hat (range)
**10Percentile**	0.78 (0.44, 0.94)	0.85 (0.77, 0.91)	0.02 (0.01, 0.02)	0.04 (0.03, 0.05)	0.03 (0.01, 0.06)	0.28 (0.23, 0.33)	0.04 (0.00, 0.07)	-24.11 (-28.17, -20.89)	31.77 (26.41, 39.22)	1.00 (1.00, 1.00)
**90Percentile**	0.92 (0.76, 0.98)	0.93 (0.88, 0.96)	0.01 (0.01, 0.01)	0.03 (0.03, 0.04)	0.03 (0.02, 0.06)	0.18 (0.15, 0.21)	0.01 (-0.02, 0.03)	-16.32 (-19.28, -13.96)	19.51 (16.23, 23.88)	1.00 (1.00, 1.00)
**Energy**	0.84 (0.24, 0.97)	0.96 (0.94, 0.98)	0.01 (0.01, 0.01)	0.06 (0.05, 0.07)	0.02 (0.01, 0.06)	0.57 (0.48, 0.69)	-0.02 (-0.09, 0.05)	-43.50 (-49.64, -38.26)	76.98 (61.97, 98.55)	1.00 (1.00, 1.01)
**Entropy**	0.80 (0.54, 0.94)	0.75 (0.61, 0.85)	0.11 (0.10, 0.14)	0.18 (0.14, 0.23)	0.20 (0.11, 0.41)	0.26 (0.22, 0.31)	-0.01 (-0.04, 0.02)	-22.83 (-26.87, -19.74)	29.58 (24.60, 36.74)	1.00 (1.00, 1.00)
**InterquartileRange**	0.81 (0.57, 0.94)	0.74 (0.60, 0.83)	0.04 (0.03, 0.05)	0.07 (0.05, 0.08)	0.08 (0.05, 0.15)	0.57 (0.48, 0.69)	-0.05 (-0.12, 0.03)	-43.40 (-49.78, -38.17)	76.69 (61.73, 99.13)	1.00 (1.00, 1.00)
**Kurtosis**	0.61 (0.13, 0.89)	0.79 (0.67, 0.87)	0.18 (0.15, 0.22)	0.31 (0.24, 0.41)	0.19 (0.06, 0.44)	0.60 (0.51, 0.73)	0.11 (0.04, 0.19)	-45.26 (-51.74, -40.02)	82.67 (66.73, 107.23)	1.00 (1.00, 1.01)
**Maximum**	0.73 (0.35, 0.92)	0.85 (0.76, 0.91)	0.02 (0.01, 0.02)	0.04 (0.03, 0.05)	0.03 (0.01, 0.05)	0.31 (0.27, 0.38)	0.03 (-0.01, 0.07)	-26.93 (-31.48, -23.35)	36.85 (30.47, 45.95)	1.00 (1.00, 1.00)
**Mean**	0.93 (0.75, 0.98)	0.95 (0.91, 0.97)	0.01 (0.01, 0.01)	0.03 (0.03, 0.04)	0.03 (0.01, 0.06)	0.15 (0.12, 0.17)	0.01 (-0.00, 0.03)	-13.55 (-16.00, -11.63)	15.67 (13.16, 19.04)	1.00 (1.00, 1.00)
**MeanAbsoluteDeviation**	0.80 (0.53, 0.94)	0.75 (0.62, 0.85)	0.04 (0.03, 0.05)	0.07 (0.06, 0.09)	0.08 (0.04, 0.15)	0.53 (0.45, 0.64)	-0.01 (-0.08, 0.05)	-41.14 (-47.06, -36.15)	69.90 (56.62, 88.90)	1.00 (1.00, 1.00)
**Median**	0.93 (0.76, 0.98)	0.95 (0.92, 0.97)	0.01 (0.01, 0.01)	0.03 (0.03, 0.04)	0.03 (0.01, 0.06)	0.14 (0.12, 0.17)	0.01 (-0.01, 0.03)	-13.47 (-15.93, -11.54)	15.56 (13.04, 18.95)	1.00 (1.00, 1.00)
**Minimum**	0.40 (0.03, 0.77)	0.64 (0.45, 0.78)	1.44 (0.62, 33.25)	0.61 (0.43, 1.75)	0.31 (0.07, 0.77)	448.29 (381.33, 541.89)	35.40 (-21.76, 90.23)	N/A	N/A	1.00 (1.00, 1.01)
**Range**	0.68 (0.35, 0.90)	0.74 (0.61, 0.84)	0.03 (0.03, 0.04)	0.05 (0.04, 0.07)	0.05 (0.02, 0.09)	0.61 (0.52, 0.73)	0.02 (-0.05, 0.10)	-45.59 (-51.67, -40.34)	83.81 (67.62, 106.91)	1.00 (1.00, 1.00)
**RobustMeanAbsoluteDeviation**	0.82 (0.57, 0.94)	0.76 (0.62, 0.85)	0.05 (0.04, 0.06)	0.08 (0.06, 0.10)	0.09 (0.05, 0.18)	0.54 (0.46, 0.66)	-0.04 (-0.11, 0.03)	-41.95 (-48.18, -37.01)	72.27 (58.75, 92.97)	1.00 (1.00, 1.00)
**RootMeanSquared**	0.93 (0.75, 0.98)	0.95 (0.91, 0.97)	0.01 (0.01, 0.01)	0.03 (0.03, 0.04)	0.03 (0.01, 0.06)	0.15 (0.12, 0.18)	0.01 (-0.01, 0.03)	-13.59 (-16.14, -11.66)	15.73 (13.20, 19.25)	1.00 (1.00, 1.00)
**Skewness**	0.24 (0.01, 0.71)	0.70 (0.54, 0.81)	6.44 (1.93, 142.82)	1.57 (0.87, 26.73)	0.42 (0.08, 1.87)	1.40 (1.19, 1.69)	0.17 (-0.01, 0.35)	N/A	N/A	1.00 (1.00, 1.01)
**TotalEnergy**	0.85 (0.38, 0.97)	0.96 (0.94, 0.98)	0.01 (0.01, 0.01)	0.05 (0.04, 0.06)	0.02 (0.01, 0.05)	0.57 (0.49, 0.69)	-0.02 (-0.10, 0.05)	-43.56 (-49.79, -38.56)	77.18 (62.77, 99.18)	1.00 (1.00, 1.00)
**Uniformity**	0.80 (0.55, 0.94)	0.73 (0.59, 0.83)	0.11 (0.10, 0.14)	0.18 (0.14, 0.23)	0.20 (0.12, 0.42)	0.46 (0.39, 0.55)	0.02 (-0.03, 0.08)	-36.69 (-42.41, -31.96)	57.94 (46.98, 73.65)	1.00 (1.00, 1.00)
**Variance**	0.77 (0.50, 0.93)	0.75 (0.61, 0.85)	0.04 (0.03, 0.05)	0.07 (0.05, 0.08)	0.07 (0.04, 0.14)	1.08 (0.91, 1.31)	0.00 (-0.13, 0.14)	-66.09 (-72.94, -59.87)	194.92 (149.19, 269.55)	1.00 (1.00, 1.00)

glcm

Name	ICC inter (95% CI)	ICC intra (95% CI)	wlCV (95% CI)	blCV (95% CI)	bCV (95% CI)	RC (95% CI)	bias (95% CI)	-LoA (%) (95% CI)	+LoA (%) (95% CI)	R-hat (range)
**Autocorrelation**	0.64 (0.34, 0.88)	0.59 (0.41, 0.73)	0.17 (0.14, 0.20)	0.19 (0.14, 0.25)	0.20 (0.11, 0.41)	1.35 (1.15, 1.62)	-0.08 (-0.25, 0.10)	-74.07 (-80.13, -68.22)	285.62 (214.71, 403.23)	1.00 (1.00, 1.00)
**ClusterProminence**	0.74 (0.43, 0.92)	0.77 (0.64, 0.86)	0.23 (0.19, 0.38)	0.34 (0.26, 0.47)	0.28 (0.14, 0.61)	2.36 (2.01, 2.84)	0.09 (-0.21, 0.39)	-90.60 (-94.14, -86.58)	964.01 (644.94, 1607.36)	1.00 (1.00, 1.00)
**ClusterShade**	0.65 (0.10, 0.93)	0.95 (0.92, 0.97)	10.92 (3.25, 224.50)	6.48 (1.94, 157.69)	0.82 (0.18, 9.57)	94.63 (79.86, 113.85)	-4.92 (-17.02, 7.07)	N/A	N/A	1.00 (1.00, 1.01)
**ClusterTendency**	0.80 (0.53, 0.94)	0.76 (0.62, 0.85)	0.82 (0.38, 16.03)	0.50 (0.37, 1.41)	0.43 (0.23, 0.98)	1.13 (0.95, 1.36)	-0.02 (-0.16, 0.12)	-67.61 (-74.33, -61.39)	208.78 (158.98, 289.60)	1.00 (1.00, 1.00)
**Contrast**	0.70 (0.38, 0.91)	0.75 (0.62, 0.84)	3.39 (1.08, 73.03)	1.00 (0.60, 10.77)	0.52 (0.27, 1.38)	1.03 (0.88, 1.24)	-0.02 (-0.15, 0.11)	-64.36 (-71.06, -58.35)	180.57 (140.10, 245.54)	1.00 (1.00, 1.00)
**Correlation**	0.18 (0.01, 0.58)	0.42 (0.20, 0.61)	0.70 (0.48, 10.13)	0.40 (0.26, 0.67)	0.21 (0.05, 0.54)	0.42 (0.36, 0.51)	-0.00 (-0.06, 0.05)	N/A	N/A	1.00 (1.00, 1.01)
**DifferenceAverage**	0.70 (0.38, 0.90)	0.73 (0.59, 0.83)	4.10 (1.30, 87.84)	4.15 (1.42, 86.05)	1.47 (0.61, 17.57)	0.56 (0.47, 0.67)	-0.02 (-0.09, 0.05)	-42.63 (-48.85, -37.56)	74.30 (60.14, 95.49)	1.00 (1.00, 1.00)
**DifferenceEntropy**	0.74 (0.42, 0.92)	0.80 (0.68, 0.88)	0.20 (0.17, 0.27)	0.32 (0.26, 0.43)	0.25 (0.13, 0.54)	0.26 (0.22, 0.32)	-0.00 (-0.04, 0.03)	-23.14 (-27.24, -20.06)	30.11 (25.09, 37.44)	1.00 (1.00, 1.00)
**DifferenceVariance**	0.70 (0.37, 0.90)	0.78 (0.66, 0.86)	5.03 (1.54, 124.57)	4.97 (1.62, 107.95)	1.95 (0.70, 33.44)	0.96 (0.81, 1.15)	-0.01 (-0.14, 0.11)	-61.59 (-68.42, -55.54)	160.34 (124.92, 216.62)	1.00 (1.00, 1.00)
**Id**	0.62 (0.29, 0.87)	0.65 (0.48, 0.78)	0.18 (0.15, 0.22)	0.23 (0.18, 0.30)	0.20 (0.10, 0.41)	0.25 (0.21, 0.29)	0.01 (-0.02, 0.04)	-21.75 (-25.50, -18.79)	27.79 (23.14, 34.23)	1.00 (1.00, 1.00)
**Idm**	0.59 (0.25, 0.86)	0.65 (0.47, 0.77)	0.23 (0.19, 0.28)	0.27 (0.21, 0.36)	0.23 (0.11, 0.49)	0.34 (0.29, 0.41)	0.01 (-0.03, 0.05)	-28.66 (-33.47, -24.93)	40.17 (33.21, 50.32)	1.00 (1.00, 1.00)
**Idmn**	0.25 (0.02, 0.66)	0.62 (0.44, 0.76)	0.10 (0.08, 0.12)	0.13 (0.10, 0.16)	0.06 (0.01, 0.13)	1.02 (0.87, 1.25)	-0.06 (-0.19, 0.07)	-64.08 (-71.21, -57.99)	178.39 (138.04, 247.33)	1.00 (1.00, 1.00)
**Idn**	0.34 (0.03, 0.74)	0.67 (0.50, 0.79)	0.08 (0.06, 0.09)	0.11 (0.08, 0.14)	0.05 (0.01, 0.13)	0.50 (0.42, 0.60)	-0.03 (-0.09, 0.03)	-39.09 (-44.85, -34.32)	64.18 (52.25, 81.34)	1.00 (1.00, 1.00)
**Imc1**	0.47 (0.05, 0.83)	0.67 (0.50, 0.79)	0.13 (0.11, 0.15)	0.17 (0.13, 0.22)	0.11 (0.03, 0.26)	0.60 (0.51, 0.72)	0.03 (-0.05, 0.10)	-44.90 (-51.38, -39.72)	81.48 (65.90, 105.66)	1.00 (1.00, 1.00)
**Imc2**	0.59 (0.22, 0.87)	0.66 (0.49, 0.78)	0.61 (0.39, 8.96)	0.49 (0.36, 1.48)	0.35 (0.16, 0.85)	0.32 (0.27, 0.38)	0.01 (-0.03, 0.05)	-27.16 (-31.81, -23.44)	37.28 (30.62, 46.64)	1.00 (1.00, 1.00)
**InverseVariance**	0.27 (0.01, 0.71)	0.66 (0.50, 0.78)	0.11 (0.09, 0.13)	0.15 (0.11, 0.19)	0.06 (0.01, 0.16)	0.24 (0.20, 0.29)	-0.01 (-0.05, 0.02)	-21.03 (-24.81, -18.24)	26.64 (22.32, 32.99)	1.00 (1.00, 1.00)
**JointAverage**	0.62 (0.31, 0.86)	0.59 (0.40, 0.74)	0.17 (0.14, 0.21)	0.19 (0.15, 0.25)	0.19 (0.10, 0.40)	0.69 (0.58, 0.82)	-0.04 (-0.12, 0.05)	-49.72 (-56.10, -43.95)	98.87 (78.42, 127.77)	1.00 (1.00, 1.00)
**JointEnergy**	0.82 (0.57, 0.95)	0.82 (0.71, 0.89)	0.10 (0.09, 0.12)	0.20 (0.16, 0.26)	0.20 (0.11, 0.39)	0.71 (0.60, 0.86)	0.05 (-0.03, 0.15)	-50.75 (-57.53, -45.03)	103.03 (81.91, 135.47)	1.00 (1.00, 1.00)
**JointEntropy**	0.83 (0.60, 0.95)	0.83 (0.73, 0.90)	0.06 (0.05, 0.07)	0.12 (0.10, 0.16)	0.12 (0.07, 0.25)	0.23 (0.19, 0.27)	-0.02 (-0.05, 0.01)	-20.36 (-23.94, -17.55)	25.57 (21.29, 31.48)	1.00 (1.00, 1.00)
**MaximumProbability**	0.73 (0.43, 0.91)	0.73 (0.58, 0.83)	0.15 (0.12, 0.18)	0.22 (0.17, 0.28)	0.21 (0.11, 0.43)	0.73 (0.62, 0.89)	0.06 (-0.04, 0.15)	-52.03 (-58.90, -46.23)	108.44 (85.97, 143.31)	1.00 (1.00, 1.00)
**SumEntropy**	0.80 (0.56, 0.94)	0.75 (0.62, 0.84)	0.08 (0.07, 0.10)	0.14 (0.11, 0.18)	0.16 (0.09, 0.30)	0.24 (0.21, 0.29)	-0.02 (-0.05, 0.01)	-21.52 (-25.21, -18.56)	27.42 (22.79, 33.71)	1.00 (1.00, 1.00)
**SumSquares**	0.81 (0.55, 0.94)	0.78 (0.66, 0.86)	2.91 (0.93, 64.06)	3.05 (1.06, 56.61)	0.82 (0.41, 3.33)	1.00 (0.85, 1.20)	-0.02 (-0.14, 0.10)	-63.08 (-70.03, -57.19)	170.86 (133.61, 233.64)	1.00 (1.00, 1.00)

gldm

Name	ICC inter (95% CI)	ICC intra (95% CI)	wlCV (95% CI)	blCV (95% CI)	bCV (95% CI)	RC (95% CI)	bias (95% CI)	-LoA (%) (95% CI)	+LoA (%) (95% CI)	R-hat (range)
**DependenceEntropy**	0.64 (0.25, 0.89)	0.79 (0.67, 0.87)	0.03 (0.03, 0.04)	0.06 (0.05, 0.08)	0.04 (0.02, 0.09)	0.14 (0.12, 0.17)	-0.00 (-0.02, 0.02)	-12.79 (-15.22, -11.00)	14.67 (12.36, 17.95)	1.00 (1.00, 1.00)
**DependenceNonUniformity**	0.78 (0.20, 0.95)	0.94 (0.91, 0.96)	0.05 (0.04, 0.06)	0.18 (0.15, 0.22)	0.08 (0.02, 0.21)	0.54 (0.46, 0.65)	-0.06 (-0.12, 0.01)	-42.01 (-47.85, -36.96)	72.45 (58.64, 91.74)	1.00 (1.00, 1.00)

DependenceNonUniformityNormalized	0.65 (0.27, 0.89)	0.72 (0.57, 0.83)	0.10 (0.09, 0.12)	0.16 (0.12, 0.20)	0.13 (0.06, 0.27)	0.36 (0.31, 0.44)	-0.01 (-0.06, 0.03)	-30.38 (-35.38, -26.51)	43.63 (36.07, 54.75)	1.00 (1.00, 1.00)
DependenceVariance	0.54 (0.15, 0.85)	0.69 (0.52, 0.80)	4.96 (1.54, 96.29)	2.42 (1.02, 46.75)	0.82 (0.28, 4.89)	0.79 (0.66, 0.95)	0.01 (-0.09, 0.11)	-54.41 (-61.30, -48.44)	119.33 (93.95, 158.42)	1.00 (1.00, 1.01)
GrayLevelNonUniformity	0.80 (0.11, 0.96)	0.95 (0.92, 0.97)	0.05 (0.04, 0.06)	0.22 (0.18, 0.27)	0.10 (0.02, 0.25)	0.55 (0.46, 0.66)	-0.02 (-0.09, 0.05)	-42.06 (-48.17, -37.14)	72.58 (59.08, 92.95)	1.00 (1.00, 1.01)
GrayLevelVariance	0.77 (0.49, 0.93)	0.76 (0.62, 0.85)	2.88 (0.95, 61.74)	1.84 (0.76, 42.81)	0.66 (0.33, 2.03)	1.03 (0.87, 1.25)	0.01 (-0.12, 0.14)	-64.28 (-71.22, -58.12)	179.99 (138.76, 247.52)	1.00 (1.00, 1.00)
HighGrayLevelEmphasis	0.65 (0.34, 0.88)	0.60 (0.42, 0.75)	0.15 (0.13, 0.18)	0.17 (0.13, 0.23)	0.18 (0.10, 0.37)	1.25 (1.06, 1.50)	-0.05 (-0.21, 0.11)	-71.42 (-77.77, -65.52)	249.95 (190.07, 349.75)	1.00 (1.00, 1.00)
LargeDependenceEmphasis	0.68 (0.31, 0.90)	0.73 (0.58, 0.83)	0.12 (0.10, 0.14)	0.18 (0.15, 0.23)	0.16 (0.07, 0.34)	0.60 (0.51, 0.72)	0.02 (-0.06, 0.10)	-45.01 (-51.39, -39.91)	81.86 (66.42, 105.73)	1.00 (1.00, 1.00)
LargeDependenceHighGrayLevelEmphasis	0.32 (0.02, 0.75)	0.70 (0.53, 0.81)	0.09 (0.08, 0.11)	0.13 (0.10, 0.17)	0.06 (0.01, 0.15)	1.25 (1.06, 1.51)	-0.07 (-0.22, 0.09)	-71.24 (-77.87, -65.28)	247.70 (188.05, 351.77)	1.00 (1.00, 1.01)
LargeDependenceLowGrayLevelEmphasis	0.59 (0.27, 0.85)	0.51 (0.29, 0.68)	6.24 (1.93, 131.71)	2.47 (0.91, 50.55)	0.98 (0.47, 5.42)	1.95 (1.65, 2.35)	0.09 (-0.14, 0.33)	-85.76 (-90.46, -80.78)	602.49 (420.38, 947.73)	1.00 (1.00, 1.00)
LowGrayLevelEmphasis	0.50 (0.17, 0.81)	0.62 (0.44, 0.76)	0.23 (0.19, 0.29)	0.26 (0.19, 0.34)	0.19 (0.09, 0.39)	1.43 (1.21, 1.72)	0.03 (-0.15, 0.22)	-75.98 (-82.06, -70.13)	316.28 (234.76, 457.45)	1.00 (1.00, 1.00)
SmallDependenceEmphasis	0.68 (0.34, 0.90)	0.72 (0.57, 0.82)	0.17 (0.15, 0.21)	0.26 (0.20, 0.35)	0.22 (0.11, 0.47)	0.41 (0.35, 0.50)	-0.02 (-0.07, 0.04)	-33.89 (-39.21, -29.76)	51.26 (42.36, 64.49)	1.00 (1.00, 1.00)
SmallDependenceHighGrayLevelEmphasis	0.70 (0.40, 0.90)	0.63 (0.44, 0.76)	0.31 (0.24, 1.68)	0.30 (0.23, 0.45)	0.31 (0.17, 0.66)	1.40 (1.18, 1.70)	-0.02 (-0.20, 0.16)	-75.37 (-81.80, -69.33)	306.05 (226.05, 449.38)	1.00 (1.00, 1.01)
SmallDependenceLowGrayLevelEmphasis	0.41 (0.02, 0.80)	0.73 (0.58, 0.83)	0.12 (0.10, 0.14)	0.19 (0.15, 0.24)	0.09 (0.02, 0.22)	1.12 (0.95, 1.35)	-0.03 (-0.17, 0.11)	-67.39 (-74.06, -61.29)	206.69 (158.30, 285.53)	1.00 (1.00, 1.01)

glrlm

Name	ICC inter (95% CI)	ICC intra (95% CI)	wlCV (95% CI)	blCV (95% CI)	bCV (95% CI)	RC (95% CI)	bias (95% CI)	-LoA (%) (95% CI)	+LoA (%) (95% CI)	R-hat (range)
GrayLevelNonUniformity	0.83 (0.25, 0.97)	0.96 (0.94, 0.98)	0.05 (0.04, 0.06)	0.22 (0.18, 0.27)	0.10 (0.02, 0.25)	0.46 (0.39, 0.55)	-0.03 (-0.09, 0.03)	-36.74 (-42.18, -32.08)	58.07 (47.23, 72.95)	1.00 (1.00, 1.00)
GrayLevelNonUniformityNormalized	0.79 (0.52, 0.94)	0.72 (0.57, 0.82)	0.10 (0.09, 0.13)	0.16 (0.12, 0.20)	0.18 (0.10, 0.37)	0.44 (0.37, 0.54)	0.02 (-0.04, 0.08)	-35.86 (-41.63, -31.12)	55.90 (45.18, 71.31)	1.00 (1.00, 1.00)
GrayLevelVariance	0.75 (0.45, 0.92)	0.75 (0.60, 0.84)	2.99 (0.95, 60.72)	1.09 (0.60, 14.50)	0.57 (0.30, 1.54)	1.02 (0.86, 1.23)	0.02 (-0.11, 0.14)	-63.81 (-70.82, -57.68)	176.30 (136.31, 242.68)	1.00 (1.00, 1.00)
HighGrayLevelRunEmphasis	0.65 (0.34, 0.88)	0.61 (0.42, 0.75)	0.14 (0.12, 0.17)	0.17 (0.13, 0.22)	0.18 (0.10, 0.37)	1.23 (1.04, 1.48)	-0.04 (-0.19, 0.11)	-70.69 (-77.17, -64.74)	241.15 (183.58, 338.07)	1.00 (1.00, 1.00)
LongRunEmphasis	0.67 (0.31, 0.90)	0.79 (0.67, 0.87)	0.20 (0.17, 0.25)	0.34 (0.27, 0.46)	0.24 (0.11, 0.51)	0.34 (0.29, 0.41)	0.02 (-0.02, 0.06)	-28.95 (-33.57, -25.19)	40.75 (33.67, 50.54)	1.00 (1.00, 1.00)
LongRunHighGrayLevelEmphasis	0.53 (0.19, 0.84)	0.61 (0.41, 0.75)	0.11 (0.09, 0.13)	0.13 (0.10, 0.18)	0.11 (0.05, 0.24)	1.19 (1.00, 1.43)	-0.05 (-0.19, 0.11)	-69.56 (-76.18, -63.19)	228.51 (171.67, 319.80)	1.00 (1.00, 1.00)
LongRunLowGrayLevelEmphasis	0.58 (0.25, 0.85)	0.61 (0.43, 0.75)	1.01 (0.45, 17.13)	0.44 (0.32, 0.93)	0.34 (0.17, 0.81)	1.61 (1.37, 1.96)	0.06 (-0.14, 0.27)	-80.07 (-85.94, -74.61)	401.81 (293.90, 611.06)	1.00 (1.00, 1.00)
LowGrayLevelRunEmphasis	0.51 (0.15, 0.84)	0.64 (0.46, 0.77)	0.22 (0.18, 0.28)	0.26 (0.20, 0.34)	0.19 (0.08, 0.43)	1.38 (1.17, 1.67)	0.03 (-0.15, 0.20)	-74.78 (-81.09, -68.82)	296.45 (220.73, 428.93)	1.00 (1.00, 1.00)
RunEntropy	0.72 (0.41, 0.91)	0.75 (0.61, 0.84)	0.05 (0.04, 0.06)	0.08 (0.07, 0.10)	0.08 (0.04, 0.15)	0.16 (0.14, 0.19)	-0.00 (-0.02, 0.02)	-14.89 (-17.69, -12.78)	17.49 (14.65, 21.49)	1.00 (1.00, 1.00)
RunLengthNonUniformity	0.80 (0.13, 0.96)	0.96 (0.93, 0.97)	0.04 (0.03, 0.04)	0.17 (0.14, 0.21)	0.07 (0.01, 0.18)	0.52 (0.44, 0.62)	-0.06 (-0.12, 0.02)	-40.39 (-46.41, -35.54)	67.75 (55.14, 86.60)	1.00 (1.00, 1.01)
RunLengthNonUniformityNormalized	0.49 (0.15, 0.82)	0.69 (0.53, 0.80)	0.24 (0.20, 0.30)	0.31 (0.24, 0.40)	0.20 (0.08, 0.43)	0.20 (0.17, 0.25)	-0.01 (-0.03, 0.02)	-18.49 (-21.79, -15.88)	22.68 (18.88, 27.86)	1.00 (1.00, 1.00)
RunPercentage	0.57 (0.20, 0.86)	0.75 (0.61, 0.84)	0.23 (0.19, 0.29)	0.34 (0.26, 0.44)	0.22 (0.09, 0.47)	0.11 (0.09, 0.13)	-0.01 (-0.02, 0.01)	-10.51 (-12.52, -8.95)	11.74 (9.83, 14.31)	1.00 (1.00, 1.00)
RunVariance	0.58 (0.18, 0.86)	0.75 (0.62, 0.84)	2.56 (0.84, 61.78)	0.64 (0.47, 1.27)	0.36 (0.14, 0.84)	0.80 (0.67, 0.97)	0.02 (-0.08, 0.12)	-54.93 (-61.99, -49.06)	121.88 (96.30, 163.06)	1.00 (1.00, 1.00)
ShortRunEmphasis	0.53 (0.18, 0.83)	0.68 (0.51, 0.80)	0.27 (0.22, 0.36)	0.34 (0.26, 0.45)	0.23 (0.10, 0.49)	0.10 (0.08, 0.12)	-0.01 (-0.02, 0.01)	-9.51 (-11.37, -8.12)	10.51 (8.84, 12.83)	1.00 (1.00, 1.00)
ShortRunHighGrayLevelEmphasis	0.66 (0.35, 0.89)	0.62 (0.43, 0.75)	0.16 (0.13, 0.19)	0.19 (0.14, 0.24)	0.19 (0.11, 0.40)	1.25 (1.05, 1.50)	-0.04 (-0.19, 0.12)	-71.31 (-77.76, -65.12)	248.57 (186.72, 349.66)	1.00 (1.00, 1.00)
ShortRunLowGrayLevelEmphasis	0.51 (0.17, 0.83)	0.66 (0.48, 0.78)	0.19 (0.16, 0.24)	0.24 (0.19, 0.31)	0.17 (0.08, 0.37)	1.31 (1.11, 1.58)	0.01 (-0.15, 0.19)	-72.98 (-79.40, -67.06)	270.11 (203.58, 385.48)	1.00 (1.00, 1.00)

glszm

Name	ICC inter (95% CI)	ICC intra (95% CI)	wlCV (95% CI)	blCV (95% CI)	bCV (95% CI)	RC (95% CI)	bias (95% CI)	-LoA (%) (95% CI)	+LoA (%) (95% CI)	R-hat (range)
GrayLevelNonUniformity	0.70 (0.07, 0.95)	0.95 (0.92, 0.97)	0.05 (0.05, 0.07)	0.23 (0.19, 0.29)	0.08 (0.01, 0.22)	0.44 (0.37, 0.53)	-0.04 (-0.10, 0.01)	-35.55 (-41.19, -31.13)	55.15 (45.20, 70.04)	1.00 (1.00, 1.01)
GrayLevelNonUniformityNormalized	0.73 (0.44, 0.92)	0.68 (0.52, 0.80)	0.09 (0.08, 0.11)	0.13 (0.10, 0.17)	0.14 (0.08, 0.28)	0.44 (0.37, 0.54)	0.02 (-0.04, 0.07)	-35.86 (-41.44, -31.26)	55.92 (45.47, 70.76)	1.00 (1.00, 1.00)
GrayLevelVariance	0.68 (0.36, 0.90)	0.70 (0.54, 0.81)	1.35 (0.52, 27.73)	0.55 (0.39, 2.10)	0.41 (0.21, 0.97)	1.02 (0.87, 1.23)	0.03 (-0.10, 0.16)	-64.12 (-70.90, -58.00)	178.69 (138.09, 243.60)	1.00 (1.00, 1.00)
HighGrayLevelZoneEmphasis	0.65 (0.33, 0.88)	0.63 (0.44, 0.76)	0.13 (0.11, 0.16)	0.16 (0.12, 0.21)	0.17 (0.09, 0.33)	1.17 (0.98, 1.41)	-0.02 (-0.16, 0.13)	-68.83 (-75.59, -62.62)	220.84 (167.52, 309.68)	1.00 (1.00, 1.00)
LargeAreaEmphasis	0.74 (0.37, 0.93)	0.83 (0.73, 0.90)	0.17 (0.14, 0.21)	0.33 (0.26, 0.45)	0.23 (0.10, 0.51)	0.84 (0.71, 1.02)	0.04 (-0.06, 0.15)	-56.98 (-63.87, -50.97)	132.43 (103.97, 176.74)	1.00 (1.00, 1.00)

**LargeAreaHighGrayLevelEmphasis**	0.26 (0.01, 0.74)	0.80 (0.70, 0.88)	0.08 (0.07, 0.10)	0.16 (0.13, 0.19)	0.05 (0.01, 0.13)	1.17 (0.99, 1.40)	-0.03 (-0.18, 0.11)	-68.85 (-75.38, -62.75)	221.03 (168.43, 306.14)	1.00 (1.00, 1.02)
**LargeAreaLowGrayLevelEmphasis**	0.66 (0.36, 0.88)	0.58 (0.38, 0.73)	6.61 (2.03, 130.64)	3.93 (1.29, 75.68)	1.66 (0.68, 24.05)	2.04 (1.72, 2.45)	0.09 (-0.17, 0.36)	-86.94 (-91.36, -82.16)	665.70 (460.65, 1058.07)	1.00 (1.00, 1.00)
**LowGrayLevelZoneEmphasis**	0.57 (0.22, 0.85)	0.66 (0.49, 0.79)	0.21 (0.17, 0.26)	0.26 (0.20, 0.34)	0.20 (0.10, 0.45)	1.27 (1.07, 1.53)	0.00 (-0.16, 0.16)	-71.91 (-78.37, -65.82)	255.97 (192.60, 362.40)	1.00 (1.00, 1.00)
**SizeZoneNonUniformity**	0.75 (0.32, 0.94)	0.88 (0.82, 0.93)	0.09 (0.07, 0.10)	0.22 (0.19, 0.28)	0.14 (0.05, 0.30)	0.87 (0.73, 1.04)	-0.07 (-0.18, 0.05)	-57.96 (-64.65, -51.89)	137.85 (107.88, 182.92)	1.00 (1.00, 1.00)
**SizeZoneNonUniformityNormalized**	0.57 (0.22, 0.86)	0.61 (0.42, 0.76)	0.17 (0.14, 0.20)	0.20 (0.15, 0.26)	0.18 (0.08, 0.39)	0.42 (0.36, 0.52)	-0.01 (-0.06, 0.05)	-34.61 (-40.31, -30.18)	52.93 (43.23, 67.52)	1.00 (1.00, 1.00)
**SmallAreaEmphasis**	0.52 (0.17, 0.82)	0.57 (0.36, 0.72)	0.22 (0.19, 0.28)	0.24 (0.18, 0.33)	0.20 (0.09, 0.44)	0.26 (0.22, 0.31)	-0.01 (-0.04, 0.02)	-22.84 (-26.94, -19.70)	29.61 (24.54, 36.88)	1.00 (1.00, 1.00)
**SmallAreaHighGrayLevelEmphasis**	0.67 (0.36, 0.89)	0.65 (0.47, 0.77)	0.17 (0.14, 0.20)	0.21 (0.16, 0.27)	0.21 (0.11, 0.42)	1.24 (1.05, 1.49)	0.01 (-0.15, 0.16)	-71.04 (-77.57, -64.86)	245.34 (184.57, 345.93)	1.00 (1.00, 1.00)
**SmallAreaLowGrayLevelEmphasis**	0.50 (0.11, 0.83)	0.69 (0.53, 0.80)	0.14 (0.12, 0.17)	0.20 (0.16, 0.26)	0.14 (0.05, 0.30)	1.12 (0.95, 1.35)	-0.06 (-0.21, 0.07)	-67.45 (-74.20, -61.22)	207.18 (157.86, 287.57)	1.00 (1.00, 1.00)
**ZoneEntropy**	0.58 (0.11, 0.88)	0.84 (0.75, 0.90)	0.03 (0.02, 0.03)	0.07 (0.05, 0.08)	0.03 (0.01, 0.08)	0.12 (0.10, 0.14)	0.00 (-0.01, 0.02)	-11.19 (-13.41, -9.62)	12.60 (10.65, 15.48)	1.00 (1.00, 1.00)
**ZonePercentage**	0.71 (0.36, 0.91)	0.78 (0.66, 0.86)	0.19 (0.16, 0.26)	0.32 (0.25, 0.45)	0.25 (0.12, 0.53)	0.35 (0.29, 0.41)	-0.02 (-0.06, 0.03)	-29.21 (-33.92, -25.37)	41.27 (33.99, 51.32)	1.00 (1.00, 1.00)
**ZoneVariance**	0.72 (0.30, 0.92)	0.84 (0.74, 0.90)	2.64 (0.85, 55.53)	1.05 (0.65, 16.07)	0.49 (0.19, 1.39)	1.15 (0.98, 1.39)	0.06 (-0.09, 0.20)	-68.27 (-75.05, -62.30)	215.12 (165.24, 300.74)	1.00 (1.00, 1.00)

ngtdm

Name	ICC inter (95% CI)	ICC intra (95% CI)	wlCV (95% CI)	blCV (95% CI)	bCV (95% CI)	RC (95% CI)	bias (95% CI)	-LoA (%) (95% CI)	+LoA (%) (95% CI)	R-hat (range)
**Busyness**	0.53 (0.14, 0.84)	0.57 (0.36, 0.72)	8.08 (2.41, 164.49)	5.37 (1.63, 117.69)	2.29 (0.75, 52.14)	1.26 (1.07, 1.51)	-0.01 (-0.17, 0.15)	-71.71 (-78.01, -65.65)	253.46 (191.11, 354.73)	1.00 (1.00, 1.00)
**Coarseness**	0.79 (0.18, 0.96)	0.96 (0.94, 0.98)	0.05 (0.04, 0.06)	0.24 (0.20, 0.29)	0.09 (0.02, 0.23)	0.49 (0.41, 0.59)	0.02 (-0.04, 0.08)	-38.60 (-44.56, -33.73)	62.86 (50.90, 80.38)	1.00 (1.00, 1.01)
**Complexity**	0.70 (0.36, 0.91)	0.78 (0.65, 0.86)	0.16 (0.14, 0.20)	0.27 (0.22, 0.35)	0.21 (0.10, 0.44)	1.40 (1.18, 1.69)	0.06 (-0.11, 0.24)	-75.29 (-81.51, -69.31)	304.70 (225.80, 440.97)	1.00 (1.00, 1.00)
**Contrast**	0.62 (0.24, 0.88)	0.74 (0.60, 0.83)	0.12 (0.10, 0.14)	0.19 (0.15, 0.24)	0.14 (0.06, 0.31)	0.91 (0.77, 1.09)	-0.03 (-0.14, 0.09)	-59.70 (-66.52, -53.75)	148.15 (116.20, 198.69)	1.00 (1.00, 1.00)
**Strength**	0.40 (0.04, 0.80)	0.70 (0.54, 0.81)	6.37 (1.91, 152.03)	9.60 (2.50, 208.56)	2.84 (0.66, 62.63)	1.25 (1.06, 1.51)	0.13 (-0.02, 0.29)	-71.40 (-77.98, -65.46)	249.69 (189.51, 354.07)	1.00 (1.00, 1.01)

shape

Name	ICC inter (95% CI)	ICC intra (95% CI)	wlCV (95% CI)	blCV (95% CI)	bCV (95% CI)	RC (95% CI)	bias (95% CI)	-LoA (%) (95% CI)	+LoA (%) (95% CI)	R-hat (range)
**Elongation**	0.38 (0.02, 0.82)	0.86 (0.78, 0.92)	0.50 (0.34, 5.40)	0.64 (0.49, 1.02)	0.19 (0.03, 0.55)	0.24 (0.21, 0.29)	0.01 (-0.02, 0.04)	-21.66 (-25.28, -18.65)	27.65 (22.93, 33.84)	1.00 (1.00, 1.00)
**Flatness**	0.37 (0.02, 0.83)	0.85 (0.76, 0.91)	0.20 (0.16, 0.26)	0.37 (0.29, 0.48)	0.12 (0.02, 0.34)	0.30 (0.25, 0.36)	-0.02 (-0.06, 0.02)	-25.96 (-30.46, -22.44)	35.05 (28.94, 43.80)	1.00 (1.00, 1.00)
**LeastAxisLength**	0.33 (0.02, 0.85)	0.92 (0.87, 0.95)	0.04 (0.03, 0.04)	0.12 (0.10, 0.15)	0.03 (0.00, 0.09)	0.27 (0.23, 0.33)	-0.03 (-0.07, 0.00)	-23.80 (-27.95, -20.57)	31.24 (25.89, 38.79)	1.00 (1.00, 1.02)
**MajorAxisLength**	0.76 (0.08, 0.95)	0.94 (0.90, 0.96)	0.03 (0.02, 0.03)	0.11 (0.09, 0.13)	0.05 (0.01, 0.12)	0.26 (0.22, 0.31)	-0.01 (-0.05, 0.02)	-23.08 (-26.94, -19.93)	30.00 (24.89, 36.88)	1.00 (1.00, 1.01)
**Maximum2DDiameterColumn**	0.47 (0.03, 0.89)	0.94 (0.90, 0.96)	0.03 (0.02, 0.03)	0.11 (0.09, 0.13)	0.03 (0.01, 0.08)	0.26 (0.22, 0.31)	-0.02 (-0.06, 0.01)	-22.83 (-26.85, -19.73)	29.58 (24.58, 36.71)	1.00 (1.00, 1.00)
**Maximum2DDiameterRow**	0.66 (0.06, 0.93)	0.94 (0.90, 0.96)	0.03 (0.02, 0.03)	0.11 (0.09, 0.13)	0.04 (0.01, 0.10)	0.26 (0.22, 0.31)	0.00 (-0.03, 0.04)	-23.04 (-26.98, -19.95)	29.94 (24.93, 36.95)	1.00 (1.00, 1.01)
**Maximum2DDiameterSlice**	0.77 (0.12, 0.95)	0.94 (0.90, 0.96)	0.03 (0.02, 0.03)	0.10 (0.08, 0.13)	0.05 (0.01, 0.12)	0.24 (0.20, 0.29)	-0.02 (-0.05, 0.01)	-21.36 (-25.22, -18.41)	27.16 (22.57, 33.72)	1.00 (1.00, 1.00)
**Maximum3DDiameter**	0.73 (0.07, 0.95)	0.94 (0.90, 0.96)	0.03 (0.02, 0.03)	0.10 (0.09, 0.13)	0.04 (0.01, 0.11)	0.25 (0.21, 0.31)	-0.01 (-0.04, 0.02)	-22.35 (-26.31, -19.27)	28.78 (23.88, 35.71)	1.00 (1.00, 1.01)
**MeshVolume**	0.79 (0.15, 0.96)	0.96 (0.94, 0.98)	0.02 (0.02, 0.03)	0.11 (0.09, 0.13)	0.04 (0.01, 0.11)	0.49 (0.42, 0.60)	-0.05 (-0.11, 0.02)	-39.02 (-45.21, -34.16)	63.98 (51.88, 82.51)	1.00 (1.00, 1.00)
**MinorAxisLength**	0.70 (0.07, 0.94)	0.95 (0.91, 0.97)	0.03 (0.02, 0.03)	0.11 (0.09, 0.13)	0.04 (0.01, 0.10)	0.21 (0.18, 0.26)	-0.00 (-0.03, 0.02)	-19.21 (-22.75, -16.57)	23.78 (19.86, 29.45)	1.00 (1.00, 1.01)
**Sphericity**	0.17 (0.01, 0.73)	0.87 (0.79, 0.92)	0.15 (0.13, 0.18)	0.34 (0.27, 0.43)	0.06 (0.01, 0.22)	0.12 (0.10, 0.14)	0.00 (-0.01, 0.02)	-11.28 (-13.34, -9.62)	12.71 (10.65, 15.40)	1.00 (1.00, 1.01)
**SurfaceArea**	0.76 (0.14, 0.96)	0.96 (0.93, 0.98)	0.02 (0.02, 0.02)	0.09 (0.07, 0.11)	0.03 (0.01, 0.09)	0.38 (0.33, 0.46)	-0.03 (-0.08, 0.02)	-31.86 (-37.17, -27.84)	46.76 (38.58, 59.16)	1.00 (1.00, 1.01)
**SurfaceVolumeRatio**	0.64 (0.04, 0.94)	0.95 (0.92, 0.97)	0.07 (0.06, 0.09)	0.29 (0.24, 0.36)	0.09 (0.01, 0.26)	0.17 (0.14, 0.21)	0.01 (-0.01, 0.04)	-15.75 (-18.54, -13.43)	18.70 (15.52, 22.76)	1.00 (1.00, 1.01)
**VoxelVolume**	0.80 (0.21, 0.96)	0.96 (0.94, 0.98)	0.02 (0.02, 0.02)	0.11 (0.09, 0.13)	0.04 (0.01, 0.10)	0.48 (0.40, 0.58)	-0.04 (-0.10, 0.02)	-37.97 (-43.78, -33.20)	61.21 (49.69, 77.87)	1.00 (1.00, 1.01)
