# Supplementary material for: Inter- and Intra-Patient Repeatability of Radiomic Features from Multiparametric Whole-Body MRI in Patients with Metastatic Prostate Cancer
Source: Cancers (Basel). 2024 Apr 25;16(9):1647. doi: 10.3390/cancers16091647 (PMC11083580; doi:10.3390/cancers16091647)
Supplement: Supplementary file 1 [file cancers-16-01647-s001.zip › Supplementary S5.pdf]

firstorder

Name	ICC inter (95% CI)	ICC intra (95% CI)	wlCV (95% CI)	blCV (95% CI)	bCV (95% CI)	RC (95% CI)	bias (95% CI)	-LoA (%) (95% CI)	+LoA (%) (95% CI)	R-hat (range)
**10Percentile**	0.52 (0.05, 0.90)	0.83 (0.71, 0.90)	0.20 (0.16, 0.27)	0.36 (0.27, 0.51)	0.16 (0.03, 0.45)	0.49 (0.41, 0.61)	-0.01 (-0.08, 0.07)	-38.97 (-45.92, -33.48)	63.84 (50.32, 84.91)	1.00 (1.00, 1.01)
**90Percentile**	0.68 (0.14, 0.93)	0.87 (0.77, 0.93)	0.04 (0.03, 0.05)	0.10 (0.08, 0.13)	0.06 (0.02, 0.13)	0.29 (0.24, 0.36)	0.02 (-0.02, 0.07)	-25.13 (-30.24, -21.27)	33.56 (27.01, 43.36)	1.00 (1.00, 1.00)
**Energy**	0.41 (0.04, 0.89)	0.88 (0.78, 0.93)	0.03 (0.02, 0.04)	0.07 (0.06, 0.09)	0.02 (0.01, 0.08)	0.83 (0.68, 1.04)	0.03 (-0.10, 0.15)	-56.26 (-64.65, -49.24)	128.60 (96.99, 182.87)	1.00 (1.00, 1.01)
**Entropy**	0.66 (0.14, 0.92)	0.84 (0.72, 0.91)	0.07 (0.06, 0.09)	0.15 (0.12, 0.19)	0.09 (0.03, 0.22)	0.16 (0.13, 0.21)	0.01 (-0.02, 0.03)	-15.07 (-18.57, -12.58)	17.74 (14.39, 22.81)	1.00 (1.00, 1.00)
**InterquartileRange**	0.59 (0.15, 0.90)	0.81 (0.67, 0.89)	0.07 (0.06, 0.09)	0.15 (0.11, 0.19)	0.09 (0.03, 0.21)	0.36 (0.29, 0.45)	0.03 (-0.02, 0.09)	-29.98 (-36.01, -25.53)	42.82 (34.27, 56.27)	1.00 (1.00, 1.00)
**Kurtosis**	0.09 (0.00, 0.51)	0.25 (0.02, 0.51)	0.29 (0.24, 0.37)	0.16 (0.05, 0.26)	0.09 (0.02, 0.27)	1.33 (1.10, 1.65)	0.00 (-0.20, 0.20)	-73.66 (-80.78, -66.68)	279.69 (200.09, 420.27)	1.00 (1.00, 1.01)
**Maximum**	0.26 (0.01, 0.70)	0.32 (0.04, 0.57)	0.09 (0.07, 0.11)	0.06 (0.02, 0.09)	0.05 (0.01, 0.13)	0.85 (0.70, 1.06)	0.02 (-0.10, 0.15)	-57.38 (-65.20, -50.45)	134.62 (101.81, 187.36)	1.00 (1.00, 1.01)
**Mean**	0.70 (0.14, 0.94)	0.90 (0.82, 0.94)	0.05 (0.04, 0.06)	0.14 (0.11, 0.18)	0.07 (0.02, 0.18)	0.27 (0.22, 0.34)	0.01 (-0.03, 0.05)	-23.87 (-28.87, -20.09)	31.36 (25.14, 40.59)	1.00 (1.00, 1.00)
**MeanAbsoluteDeviation**	0.56 (0.09, 0.88)	0.77 (0.61, 0.87)	0.11 (0.09, 0.13)	0.18 (0.14, 0.24)	0.11 (0.03, 0.28)	0.37 (0.31, 0.47)	0.02 (-0.03, 0.08)	-31.08 (-37.24, -26.32)	45.10 (35.72, 59.35)	1.00 (1.00, 1.00)
**Median**	0.71 (0.10, 0.95)	0.92 (0.87, 0.96)	0.05 (0.04, 0.06)	0.16 (0.12, 0.20)	0.07 (0.01, 0.19)	0.25 (0.21, 0.32)	0.02 (-0.02, 0.05)	-22.42 (-27.10, -18.86)	28.91 (23.24, 37.18)	1.00 (1.00, 1.01)
**Minimum**	0.07 (0.00, 0.50)	0.38 (0.08, 0.60)	16.42 (4.59, 348.75)	4.31 (1.06, 117.49)	0.81 (0.14, 13.91)	2.44 (2.00, 3.04)	0.17 (-0.21, 0.50)	N/A	N/A	1.01 (1.00, 1.02)
**Range**	0.25 (0.01, 0.71)	0.33 (0.03, 0.58)	0.09 (0.07, 0.11)	0.06 (0.02, 0.09)	0.05 (0.01, 0.13)	0.85 (0.69, 1.05)	0.02 (-0.11, 0.14)	-57.11 (-65.01, -49.83)	133.13 (99.31, 185.82)	1.00 (1.00, 1.01)
**RobustMeanAbsoluteDeviation**	0.60 (0.10, 0.91)	0.82 (0.70, 0.90)	0.15 (0.12, 0.19)	0.28 (0.21, 0.38)	0.15 (0.04, 0.40)	0.33 (0.28, 0.42)	0.02 (-0.03, 0.07)	-28.38 (-34.28, -24.10)	39.63 (31.75, 52.16)	1.00 (1.00, 1.00)
**RootMeanSquared**	0.65 (0.09, 0.92)	0.86 (0.75, 0.92)	0.05 (0.04, 0.06)	0.12 (0.09, 0.16)	0.07 (0.02, 0.16)	0.31 (0.26, 0.39)	0.02 (-0.03, 0.06)	-26.72 (-32.18, -22.55)	36.46 (29.11, 47.45)	1.00 (1.00, 1.00)
**Skewness**	0.05 (0.00, 0.45)	0.26 (0.04, 0.52)	0.63 (0.48, 2.97)	0.30 (0.12, 0.51)	0.12 (0.02, 0.44)	1.79 (1.47, 2.19)	-0.04 (-0.29, 0.22)	N/A	N/A	1.00 (1.00, 1.01)
**TotalEnergy**	0.37 (0.01, 0.88)	0.88 (0.78, 0.93)	0.02 (0.02, 0.03)	0.06 (0.05, 0.08)	0.02 (0.00, 0.06)	0.82 (0.68, 1.03)	0.03 (-0.09, 0.15)	-56.17 (-64.33, -49.49)	128.17 (97.97, 180.33)	1.00 (1.00, 1.01)
**Uniformity**	0.63 (0.10, 0.91)	0.86 (0.75, 0.92)	0.07 (0.05, 0.08)	0.15 (0.12, 0.20)	0.08 (0.02, 0.19)	0.27 (0.22, 0.34)	-0.01 (-0.06, 0.03)	-23.64 (-28.81, -19.95)	30.97 (24.92, 40.48)	1.00 (1.00, 1.00)
**Variance**	0.47 (0.07, 0.84)	0.68 (0.47, 0.82)	0.11 (0.09, 0.13)	0.15 (0.11, 0.20)	0.10 (0.03, 0.24)	0.91 (0.75, 1.13)	0.04 (-0.09, 0.18)	-59.71 (-67.85, -52.69)	148.23 (111.37, 211.01)	1.00 (1.00, 1.00)

glcm

Name	ICC inter (95% CI)	ICC intra (95% CI)	wlCV (95% CI)	blCV (95% CI)	bCV (95% CI)	RC (95% CI)	bias (95% CI)	-LoA (%) (95% CI)	+LoA (%) (95% CI)	R-hat (range)
**Autocorrelation**	0.68 (0.12, 0.94)	0.90 (0.81, 0.94)	0.08 (0.06, 0.10)	0.21 (0.17, 0.27)	0.10 (0.03, 0.27)	0.45 (0.37, 0.56)	0.01 (-0.06, 0.07)	-36.14 (-42.98, -30.94)	56.59 (44.80, 75.38)	1.00 (1.00, 1.01)
**ClusterProminence**	0.28 (0.03, 0.71)	0.44 (0.14, 0.66)	0.20 (0.17, 0.26)	0.17 (0.09, 0.25)	0.12 (0.03, 0.30)	2.61 (2.14, 3.28)	0.05 (-0.34, 0.45)	-92.62 (-96.24, -88.29)	1255.62 (753.94, 2561.16)	1.00 (1.00, 1.00)
**ClusterShade**	0.08 (0.00, 0.43)	0.12 (0.01, 0.39)	7.43 (1.45, 199.81)	0.75 (0.16, 12.88)	0.42 (0.09, 1.83)	81.40 (67.64, 97.13)	4.96 (-7.03, 17.42)	N/A	N/A	1.00 (1.00, 1.01)
**ClusterTendency**	0.50 (0.10, 0.84)	0.67 (0.46, 0.81)	0.22 (0.18, 0.29)	0.27 (0.20, 0.38)	0.18 (0.06, 0.44)	0.84 (0.69, 1.05)	0.05 (-0.07, 0.17)	-56.93 (-65.18, -49.92)	132.19 (99.67, 187.19)	1.00 (1.00, 1.00)
**Contrast**	0.64 (0.17, 0.91)	0.76 (0.60, 0.87)	1.34 (0.52, 27.05)	0.57 (0.40, 2.76)	0.35 (0.12, 0.96)	0.78 (0.65, 0.98)	0.04 (-0.08, 0.15)	-54.37 (-62.43, -47.63)	119.16 (90.95, 166.20)	1.00 (1.00, 1.00)
**Correlation**	0.20 (0.01, 0.63)	0.39 (0.09, 0.62)	0.85 (0.49, 14.09)	0.37 (0.17, 0.61)	0.22 (0.05, 0.59)	0.32 (0.27, 0.41)	0.01 (-0.04, 0.06)	N/A	N/A	1.00 (1.00, 1.01)
**DifferenceAverage**	0.66 (0.20, 0.92)	0.80 (0.65, 0.89)	3.68 (1.17, 78.15)	3.34 (1.21, 89.29)	0.90 (0.31, 7.72)	0.40 (0.33, 0.50)	0.01 (-0.05, 0.07)	-32.96 (-39.31, -27.93)	49.16 (38.75, 64.76)	1.00 (1.00, 1.00)
**DifferenceEntropy**	0.69 (0.17, 0.93)	0.79 (0.64, 0.88)	0.12 (0.10, 0.15)	0.21 (0.16, 0.28)	0.16 (0.05, 0.38)	0.20 (0.16, 0.24)	0.01 (-0.02, 0.04)	-17.72 (-21.55, -14.88)	21.54 (17.48, 27.47)	1.00 (1.00, 1.01)
**DifferenceVariance**	0.61 (0.19, 0.89)	0.70 (0.50, 0.83)	5.78 (1.82, 121.33)	3.83 (1.27, 81.67)	1.37 (0.49, 23.64)	0.78 (0.64, 0.99)	0.05 (-0.06, 0.17)	-54.24 (-62.84, -47.24)	118.52 (89.55, 169.14)	1.00 (1.00, 1.00)
**Id**	0.61 (0.08, 0.90)	0.80 (0.66, 0.88)	0.12 (0.09, 0.14)	0.22 (0.17, 0.28)	0.13 (0.03, 0.33)	0.17 (0.14, 0.21)	-0.00 (-0.03, 0.02)	-15.52 (-18.99, -12.95)	18.37 (14.88, 23.44)	1.00 (1.00, 1.00)
**Idm**	0.58 (0.11, 0.89)	0.79 (0.64, 0.88)	0.15 (0.12, 0.19)	0.26 (0.20, 0.34)	0.16 (0.05, 0.37)	0.24 (0.20, 0.30)	-0.00 (-0.04, 0.04)	-21.60 (-26.00, -18.15)	27.55 (22.17, 35.14)	1.00 (1.00, 1.00)
**Idmn**	0.18 (0.01, 0.61)	0.39 (0.11, 0.61)	0.12 (0.10, 0.15)	0.09 (0.05, 0.14)	0.06 (0.01, 0.15)	1.26 (1.03, 1.58)	0.02 (-0.18, 0.20)	-71.71 (-79.43, -64.12)	253.49 (178.74, 386.04)	1.00 (1.00, 1.01)
**Idn**	0.21 (0.02, 0.64)	0.44 (0.18, 0.65)	0.10 (0.08, 0.12)	0.08 (0.05, 0.12)	0.05 (0.01, 0.12)	0.63 (0.51, 0.78)	0.00 (-0.09, 0.10)	-46.62 (-54.04, -40.17)	87.34 (67.14, 117.57)	1.00 (1.00, 1.00)
**Imc1**	0.12 (0.00, 0.63)	0.69 (0.51, 0.83)	0.12 (0.10, 0.14)	0.17 (0.13, 0.22)	0.04 (0.01, 0.14)	0.71 (0.58, 0.88)	-0.03 (-0.14, 0.07)	-50.63 (-58.40, -44.12)	102.57 (78.96, 140.37)	1.00 (1.00, 1.00)
**Imc2**	0.13 (0.01, 0.63)	0.64 (0.42, 0.79)	0.35 (0.26, 2.73)	0.33 (0.24, 0.47)	0.10 (0.02, 0.32)	0.38 (0.31, 0.47)	-0.02 (-0.07, 0.04)	-31.49 (-37.73, -26.68)	45.96 (36.39, 60.58)	1.00 (1.00, 1.01)
**InverseVariance**	0.26 (0.01, 0.71)	0.58 (0.33, 0.74)	0.11 (0.09, 0.13)	0.12 (0.08, 0.16)	0.06 (0.01, 0.16)	0.23 (0.19, 0.29)	0.01 (-0.03, 0.04)	-20.71 (-25.24, -17.44)	26.11 (21.12, 33.76)	1.00 (1.00, 1.02)
**JointAverage**	0.68 (0.08, 0.94)	0.90 (0.83, 0.95)	0.08 (0.07, 0.10)	0.22 (0.18, 0.29)	0.10 (0.02, 0.28)	0.22 (0.18, 0.27)	0.00 (-0.03, 0.03)	-19.78 (-23.98, -16.72)	24.66 (20.07, 31.55)	1.00 (1.00, 1.00)
**JointEnergy**	0.64 (0.11, 0.92)	0.84 (0.72, 0.91)	0.07 (0.06, 0.09)	0.15 (0.12, 0.20)	0.09 (0.02, 0.23)	0.51 (0.42, 0.64)	-0.03 (-0.11, 0.05)	-40.16 (-47.40, -34.59)	67.11 (52.89, 90.12)	1.00 (1.00, 1.00)
**JointEntropy**	0.69 (0.20, 0.93)	0.83 (0.70, 0.90)	0.04 (0.03, 0.05)	0.09 (0.07, 0.11)	0.06 (0.02, 0.14)	0.16 (0.14, 0.21)	0.01 (-0.01, 0.03)	-15.17 (-18.70, -12.63)	17.89 (14.46, 23.01)	1.00 (1.00, 1.00)
**MaximumProbability**	0.39 (0.03, 0.83)	0.73 (0.55, 0.85)	0.11 (0.09, 0.14)	0.17 (0.13, 0.23)	0.08 (0.02, 0.23)	0.62 (0.51, 0.78)	-0.02 (-0.11, 0.08)	-46.23 (-54.20, -40.03)	85.99 (66.74, 118.33)	1.00 (1.00, 1.01)
**SumEntropy**	0.63 (0.14, 0.91)	0.76 (0.59, 0.86)	0.05 (0.04, 0.06)	0.09 (0.07, 0.12)	0.07 (0.02, 0.15)	0.15 (0.13, 0.19)	0.01 (-0.01, 0.04)	-14.09 (-17.31, -11.78)	16.40 (13.36, 20.93)	1.00 (1.00, 1.00)
**SumSquares**	0.57 (0.09, 0.88)	0.72 (0.54, 0.84)	2.60 (0.89, 53.43)	0.87 (0.54, 8.46)	0.44 (0.13, 1.38)	0.78 (0.64, 1.01)	0.04 (-0.07, 0.16)	-54.00 (-63.65, -47.08)	117.41 (88.98, 175.08)	1.00 (1.00, 1.01)

gldm

Name	ICC inter (95% CI)	ICC intra (95% CI)	wlCV (95% CI)	blCV (95% CI)	bCV (95% CI)	RC (95% CI)	bias (95% CI)	-LoA (%) (95% CI)	+LoA (%) (95% CI)	R-hat (range)
**DependenceEntropy**	0.39 (0.01, 0.83)	0.74 (0.56, 0.85)	0.02 (0.02, 0.03)	0.04 (0.03, 0.05)	0.02 (0.00, 0.05)	0.09 (0.07, 0.11)	-0.00 (-0.01, 0.01)	-8.39 (-10.32, -6.94)	9.16 (7.46, 11.50)	1.00 (1.00, 1.01)
**DependenceNonUniformity**	0.49 (0.04, 0.93)	0.94 (0.89, 0.97)	0.04 (0.03, 0.05)	0.15 (0.12, 0.19)	0.04 (0.01, 0.14)	0.51 (0.42, 0.63)	0.01 (-0.07, 0.08)	-39.71 (-46.82, -33.97)	65.87 (51.45, 88.03)	1.00 (1.00, 1.00)

**DependenceNonUniformityNormalized**	0.65 (0.06, 0.92)	0.88 (0.80, 0.93)	0.06 (0.05, 0.08)	0.16 (0.13, 0.21)	0.08 (0.01, 0.19)	0.23 (0.19, 0.28)	0.01 (-0.02, 0.04)	-20.17 (-24.64, -16.92)	25.27 (20.36, 32.70)	1.00 (1.00, 1.01)
**DependenceVariance**	0.53 (0.06, 0.88)	0.82 (0.68, 0.90)	2.95 (0.95, 55.03)	1.57 (0.80, 28.48)	0.48 (0.12, 1.92)	0.52 (0.43, 0.66)	0.00 (-0.08, 0.08)	-40.79 (-48.37, -34.97)	68.89 (53.78, 93.68)	1.00 (1.00, 1.00)
**GrayLevelNonUniformity**	0.81 (0.13, 0.97)	0.97 (0.95, 0.98)	0.04 (0.03, 0.05)	0.22 (0.18, 0.29)	0.08 (0.01, 0.23)	0.49 (0.40, 0.61)	-0.02 (-0.09, 0.05)	-38.64 (-45.49, -33.29)	62.97 (49.90, 83.45)	1.00 (1.00, 1.01)
**GrayLevelVariance**	0.45 (0.02, 0.84)	0.68 (0.48, 0.81)	1.69 (0.64, 40.03)	0.60 (0.41, 1.84)	0.32 (0.05, 0.89)	0.89 (0.74, 1.12)	0.04 (-0.09, 0.17)	-58.89 (-67.44, -52.25)	143.24 (109.41, 207.15)	1.00 (1.00, 1.02)
**HighGrayLevelEmphasis**	0.68 (0.11, 0.93)	0.87 (0.77, 0.93)	0.08 (0.06, 0.10)	0.19 (0.15, 0.25)	0.10 (0.03, 0.25)	0.50 (0.41, 0.62)	0.01 (-0.06, 0.09)	-39.47 (-46.38, -33.81)	65.19 (51.09, 86.50)	1.00 (1.00, 1.00)
**LargeDependenceEmphasis**	0.70 (0.09, 0.94)	0.91 (0.83, 0.95)	0.07 (0.05, 0.08)	0.19 (0.15, 0.25)	0.10 (0.02, 0.24)	0.34 (0.28, 0.43)	-0.01 (-0.06, 0.05)	-28.83 (-34.99, -24.52)	40.50 (32.49, 53.83)	1.00 (1.00, 1.01)
**LargeDependenceHighGrayLevelEmphasis**	0.63 (0.14, 0.91)	0.79 (0.64, 0.88)	0.03 (0.03, 0.04)	0.07 (0.05, 0.09)	0.04 (0.01, 0.10)	0.39 (0.32, 0.49)	0.01 (-0.05, 0.07)	-32.59 (-38.72, -27.56)	48.34 (38.04, 63.18)	1.00 (1.00, 1.00)
**LargeDependenceLowGrayLevelEmphasis**	0.62 (0.05, 0.93)	0.92 (0.86, 0.96)	2.00 (0.69, 38.58)	3.03 (1.24, 69.25)	0.63 (0.10, 4.72)	0.70 (0.57, 0.87)	0.04 (-0.07, 0.14)	-50.16 (-57.95, -43.72)	100.64 (77.69, 137.80)	1.00 (1.00, 1.02)
**LowGrayLevelEmphasis**	0.62 (0.08, 0.93)	0.90 (0.82, 0.95)	0.11 (0.09, 0.14)	0.29 (0.23, 0.39)	0.12 (0.03, 0.34)	0.40 (0.33, 0.50)	0.04 (-0.01, 0.10)	-33.00 (-39.47, -28.23)	49.26 (39.34, 65.22)	1.00 (1.00, 1.01)
**SmallDependenceEmphasis**	0.75 (0.23, 0.95)	0.91 (0.85, 0.95)	0.10 (0.08, 0.12)	0.28 (0.22, 0.37)	0.15 (0.05, 0.35)	0.23 (0.19, 0.29)	0.00 (-0.03, 0.04)	-20.50 (-24.82, -17.28)	25.79 (20.88, 33.02)	1.00 (1.00, 1.00)
**SmallDependenceHighGrayLevelEmphasis**	0.67 (0.18, 0.92)	0.84 (0.73, 0.91)	0.22 (0.17, 1.15)	0.35 (0.27, 0.50)	0.21 (0.07, 0.51)	0.74 (0.61, 0.93)	0.01 (-0.10, 0.12)	-52.48 (-60.61, -45.72)	110.44 (84.24, 153.85)	1.00 (1.00, 1.00)
**SmallDependenceLowGrayLevelEmphasis**	0.42 (0.01, 0.81)	0.60 (0.38, 0.77)	0.06 (0.05, 0.07)	0.07 (0.05, 0.09)	0.05 (0.01, 0.11)	0.40 (0.33, 0.50)	0.04 (-0.02, 0.09)	-32.98 (-39.30, -27.93)	49.21 (38.76, 64.74)	1.00 (1.00, 1.02)

glrlm

Name	ICC inter (95% CI)	ICC intra (95% CI)	wlCV (95% CI)	blCV (95% CI)	bCV (95% CI)	RC (95% CI)	bias (95% CI)	-LoA (%) (95% CI)	+LoA (%) (95% CI)	R-hat (range)
**GrayLevelNonUniformity**	0.78 (0.11, 0.97)	0.97 (0.94, 0.98)	0.04 (0.03, 0.05)	0.22 (0.18, 0.28)	0.07 (0.01, 0.22)	0.46 (0.38, 0.58)	-0.01 (-0.08, 0.05)	-37.06 (-43.93, -31.81)	58.87 (46.66, 78.34)	1.00 (1.00, 1.02)
**GrayLevelNonUniformityNormalized**	0.66 (0.15, 0.92)	0.85 (0.74, 0.92)	0.06 (0.05, 0.07)	0.14 (0.11, 0.18)	0.08 (0.02, 0.19)	0.26 (0.21, 0.32)	-0.01 (-0.05, 0.02)	-22.73 (-27.50, -19.22)	29.42 (23.79, 37.93)	1.00 (1.00, 1.00)
**GrayLevelVariance**	0.44 (0.06, 0.82)	0.63 (0.42, 0.78)	1.01 (0.49, 22.36)	0.50 (0.35, 1.15)	0.30 (0.08, 0.79)	0.92 (0.76, 1.15)	0.04 (-0.10, 0.18)	-60.23 (-68.27, -53.15)	151.45 (113.47, 215.13)	1.00 (1.00, 1.00)
**HighGrayLevelRunEmphasis**	0.64 (0.12, 0.92)	0.85 (0.74, 0.92)	0.08 (0.06, 0.09)	0.17 (0.13, 0.22)	0.10 (0.03, 0.24)	0.51 (0.42, 0.64)	0.01 (-0.07, 0.08)	-40.22 (-47.17, -34.26)	67.29 (52.11, 89.28)	1.00 (1.00, 1.00)
**LongRunEmphasis**	0.76 (0.14, 0.95)	0.92 (0.86, 0.96)	0.12 (0.10, 0.15)	0.36 (0.28, 0.48)	0.18 (0.04, 0.43)	0.18 (0.15, 0.22)	-0.00 (-0.03, 0.02)	-16.26 (-19.91, -13.58)	19.42 (15.71, 24.86)	1.00 (1.00, 1.01)
**LongRunHighGrayLevelEmphasis**	0.59 (0.03, 0.91)	0.85 (0.73, 0.91)	0.05 (0.04, 0.06)	0.11 (0.09, 0.14)	0.06 (0.01, 0.14)	0.39 (0.33, 0.50)	0.02 (-0.05, 0.08)	-32.59 (-39.12, -27.79)	48.35 (38.49, 64.25)	1.00 (1.00, 1.02)
**LongRunLowGrayLevelEmphasis**	0.67 (0.10, 0.94)	0.92 (0.86, 0.96)	1.79 (0.62, 31.39)	0.91 (0.63, 4.14)	0.33 (0.07, 1.08)	0.54 (0.45, 0.67)	0.04 (-0.04, 0.12)	-41.56 (-49.06, -35.98)	71.12 (56.20, 96.30)	1.00 (1.00, 1.00)
**LowGrayLevelRunEmphasis**	0.61 (0.06, 0.93)	0.90 (0.82, 0.94)	0.10 (0.08, 0.13)	0.27 (0.21, 0.36)	0.11 (0.02, 0.33)	0.38 (0.32, 0.48)	0.04 (-0.01, 0.10)	-31.85 (-38.25, -27.23)	46.73 (37.43, 61.95)	1.00 (1.00, 1.01)
**RunEntropy**	0.43 (0.05, 0.84)	0.69 (0.48, 0.82)	0.03 (0.03, 0.04)	0.05 (0.04, 0.07)	0.03 (0.01, 0.07)	0.11 (0.09, 0.14)	0.00 (-0.01, 0.02)	-10.54 (-13.16, -8.80)	11.78 (9.65, 15.16)	1.00 (1.00, 1.01)
**RunLengthNonUniformity**	0.54 (0.03, 0.94)	0.95 (0.91, 0.97)	0.03 (0.03, 0.04)	0.13 (0.11, 0.17)	0.03 (0.01, 0.12)	0.48 (0.40, 0.60)	0.00 (-0.07, 0.08)	-38.18 (-45.21, -32.79)	61.75 (48.79, 82.50)	1.00 (1.00, 1.01)
**RunLengthNonUniformityNormalized**	0.73 (0.18, 0.94)	0.92 (0.85, 0.95)	0.12 (0.10, 0.16)	0.35 (0.27, 0.47)	0.17 (0.05, 0.42)	0.10 (0.08, 0.13)	0.00 (-0.01, 0.02)	-9.63 (-11.85, -7.99)	10.66 (8.68, 13.45)	1.00 (1.00, 1.01)
**RunPercentage**	0.74 (0.16, 0.95)	0.92 (0.86, 0.96)	0.13 (0.11, 0.17)	0.38 (0.30, 0.52)	0.18 (0.05, 0.47)	0.06 (0.05, 0.08)	0.00 (-0.01, 0.01)	-5.81 (-7.33, -4.83)	6.16 (5.07, 7.91)	1.00 (1.00, 1.01)
**RunVariance**	0.76 (0.23, 0.95)	0.91 (0.84, 0.95)	0.38 (0.22, 5.55)	0.46 (0.35, 0.69)	0.24 (0.08, 0.59)	0.45 (0.37, 0.55)	0.02 (-0.05, 0.08)	-35.92 (-42.50, -30.59)	56.06 (44.06, 73.93)	1.00 (1.00, 1.00)
**ShortRunEmphasis**	0.72 (0.06, 0.95)	0.92 (0.86, 0.96)	0.13 (0.11, 0.17)	0.38 (0.29, 0.50)	0.17 (0.02, 0.46)	0.05 (0.04, 0.06)	0.00 (-0.01, 0.01)	-4.47 (-5.60, -3.74)	4.68 (3.88, 5.93)	1.00 (1.00, 1.01)
**ShortRunHighGrayLevelEmphasis**	0.65 (0.12, 0.93)	0.85 (0.74, 0.92)	0.09 (0.07, 0.11)	0.19 (0.15, 0.25)	0.11 (0.03, 0.29)	0.55 (0.45, 0.69)	0.01 (-0.07, 0.09)	-42.32 (-49.76, -36.21)	73.38 (56.76, 99.04)	1.00 (1.00, 1.00)
**ShortRunLowGrayLevelEmphasis**	0.59 (0.05, 0.91)	0.87 (0.78, 0.93)	0.08 (0.07, 0.10)	0.20 (0.16, 0.26)	0.09 (0.02, 0.26)	0.37 (0.31, 0.46)	0.05 (-0.00, 0.10)	-30.98 (-36.81, -26.37)	44.88 (35.81, 58.26)	1.00 (1.00, 1.01)

glszm

Name	ICC inter (95% CI)	ICC intra (95% CI)	wlCV (95% CI)	blCV (95% CI)	bCV (95% CI)	RC (95% CI)	bias (95% CI)	-LoA (%) (95% CI)	+LoA (%) (95% CI)	R-hat (range)
**GrayLevelNonUniformity**	0.65 (0.03, 0.96)	0.96 (0.92, 0.98)	0.05 (0.04, 0.06)	0.21 (0.17, 0.27)	0.06 (0.01, 0.21)	0.44 (0.36, 0.55)	-0.01 (-0.08, 0.05)	-35.58 (-42.52, -30.41)	55.22 (43.70, 73.96)	1.00 (1.00, 1.02)
**GrayLevelNonUniformityNormalized**	0.58 (0.08, 0.89)	0.77 (0.60, 0.87)	0.06 (0.05, 0.08)	0.11 (0.08, 0.14)	0.07 (0.02, 0.16)	0.28 (0.23, 0.36)	-0.01 (-0.06, 0.03)	-24.70 (-29.93, -20.86)	32.79 (26.36, 42.71)	1.00 (1.00, 1.00)
**GrayLevelVariance**	0.36 (0.04, 0.77)	0.52 (0.27, 0.71)	0.44 (0.33, 2.97)	0.34 (0.22, 0.55)	0.22 (0.06, 0.59)	1.02 (0.83, 1.27)	0.03 (-0.12, 0.18)	-63.82 (-71.91, -56.55)	176.36 (130.14, 256.01)	1.00 (1.00, 1.00)
**HighGrayLevelZoneEmphasis**	0.58 (0.12, 0.89)	0.77 (0.61, 0.87)	0.08 (0.06, 0.10)	0.14 (0.10, 0.18)	0.09 (0.03, 0.21)	0.58 (0.48, 0.72)	0.00 (-0.08, 0.09)	-43.80 (-51.32, -37.84)	77.93 (60.87, 105.41)	1.00 (1.00, 1.00)
**LargeAreaEmphasis**	0.76 (0.18, 0.96)	0.94 (0.89, 0.97)	0.11 (0.09, 0.14)	0.37 (0.29, 0.49)	0.16 (0.04, 0.42)	0.56 (0.46, 0.70)	-0.01 (-0.09, 0.07)	-42.92 (-50.19, -36.75)	75.20 (58.11, 100.78)	1.00 (1.00, 1.01)

**LargeAreaHighGrayLevelEmphasis**	0.66 (0.08, 0.93)	0.88 (0.79, 0.93)	0.04 (0.03, 0.05)	0.10 (0.08, 0.13)	0.05 (0.01, 0.13)	0.46 (0.38, 0.57)	0.01 (-0.06, 0.08)	-36.90 (-43.48, -31.46)	58.48 (45.91, 76.92)	1.00 (1.00, 1.01)
**LargeAreaLowGrayLevelEmphasis**	0.69 (0.10, 0.94)	0.94 (0.89, 0.97)	2.34 (0.81, 49.70)	2.89 (1.20, 60.64)	0.61 (0.13, 4.06)	0.88 (0.72, 1.10)	0.03 (-0.10, 0.15)	-58.47 (-66.87, -51.56)	140.79 (106.45, 201.80)	1.00 (1.00, 1.01)
**LowGrayLevelZoneEmphasis**	0.57 (0.06, 0.91)	0.86 (0.75, 0.92)	0.09 (0.08, 0.12)	0.22 (0.17, 0.29)	0.10 (0.02, 0.28)	0.39 (0.32, 0.48)	0.04 (-0.02, 0.10)	-32.07 (-38.31, -27.35)	47.21 (37.65, 62.10)	1.00 (1.00, 1.00)
**SizeZoneNonUniformity**	0.33 (0.03, 0.88)	0.89 (0.81, 0.94)	0.05 (0.04, 0.07)	0.15 (0.12, 0.19)	0.04 (0.01, 0.14)	0.61 (0.50, 0.77)	-0.01 (-0.10, 0.09)	-45.87 (-53.51, -39.37)	84.75 (64.94, 115.08)	1.00 (1.00, 1.02)
**SizeZoneNonUniformityNormalized**	0.60 (0.13, 0.90)	0.81 (0.68, 0.89)	0.11 (0.09, 0.14)	0.21 (0.16, 0.28)	0.12 (0.04, 0.29)	0.27 (0.22, 0.33)	-0.01 (-0.05, 0.03)	-23.44 (-28.30, -19.67)	30.61 (24.49, 39.47)	1.00 (1.00, 1.00)
**SmallAreaEmphasis**	0.53 (0.06, 0.86)	0.76 (0.60, 0.87)	0.15 (0.12, 0.19)	0.24 (0.19, 0.33)	0.14 (0.04, 0.33)	0.17 (0.14, 0.21)	-0.01 (-0.03, 0.02)	-15.24 (-18.74, -12.64)	17.98 (14.47, 23.07)	1.00 (1.00, 1.00)
**SmallAreaHighGrayLevelEmphasis**	0.55 (0.10, 0.88)	0.73 (0.55, 0.84)	0.12 (0.10, 0.14)	0.18 (0.13, 0.24)	0.12 (0.04, 0.29)	0.74 (0.61, 0.92)	-0.00 (-0.11, 0.11)	-52.33 (-60.33, -45.56)	109.75 (83.68, 152.07)	1.00 (1.00, 1.00)
**SmallAreaLowGrayLevelEmphasis**	0.31 (0.01, 0.77)	0.58 (0.32, 0.75)	0.08 (0.07, 0.11)	0.10 (0.07, 0.13)	0.06 (0.01, 0.15)	0.48 (0.39, 0.60)	0.01 (-0.06, 0.08)	-38.08 (-45.27, -32.49)	61.50 (48.13, 82.72)	1.00 (1.00, 1.01)
**ZoneEntropy**	0.38 (0.03, 0.81)	0.73 (0.54, 0.84)	0.02 (0.02, 0.03)	0.04 (0.03, 0.05)	0.02 (0.00, 0.05)	0.10 (0.08, 0.13)	0.00 (-0.01, 0.02)	-9.69 (-11.83, -8.05)	10.73 (8.76, 13.42)	1.00 (1.00, 1.00)
**ZonePercentage**	0.77 (0.16, 0.95)	0.93 (0.87, 0.96)	0.11 (0.09, 0.14)	0.34 (0.27, 0.46)	0.17 (0.04, 0.42)	0.20 (0.16, 0.25)	0.01 (-0.02, 0.04)	-18.17 (-22.21, -15.13)	22.20 (17.82, 28.55)	1.00 (1.00, 1.01)
**ZoneVariance**	0.71 (0.10, 0.94)	0.93 (0.87, 0.96)	0.73 (0.36, 13.43)	0.83 (0.59, 3.61)	0.31 (0.07, 0.92)	0.81 (0.66, 1.02)	0.02 (-0.10, 0.14)	-55.51 (-63.79, -48.34)	124.79 (93.58, 176.17)	1.00 (1.00, 1.01)

ngtdm

Name	ICC inter (95% CI)	ICC intra (95% CI)	wlCV (95% CI)	blCV (95% CI)	bCV (95% CI)	RC (95% CI)	bias (95% CI)	-LoA (%) (95% CI)	+LoA (%) (95% CI)	R-hat (range)
**Busyness**	0.57 (0.08, 0.91)	0.86 (0.75, 0.92)	1.63 (0.63, 31.94)	1.25 (0.74, 18.58)	0.43 (0.10, 1.74)	0.93 (0.76, 1.15)	0.02 (-0.12, 0.16)	-60.40 (-68.33, -53.35)	152.53 (114.37, 215.79)	1.00 (1.00, 1.00)
**Coarseness**	0.56 (0.06, 0.94)	0.94 (0.90, 0.97)	0.05 (0.04, 0.06)	0.19 (0.15, 0.25)	0.05 (0.01, 0.19)	0.56 (0.46, 0.70)	0.01 (-0.07, 0.10)	-42.97 (-50.14, -37.08)	75.36 (58.93, 100.56)	1.00 (1.00, 1.01)
**Complexity**	0.34 (0.04, 0.78)	0.40 (0.10, 0.63)	0.17 (0.14, 0.21)	0.13 (0.07, 0.20)	0.12 (0.03, 0.31)	1.79 (1.46, 2.22)	-0.00 (-0.26, 0.26)	-83.24 (-89.19, -76.85)	496.51 (331.88, 825.01)	1.00 (1.00, 1.00)
**Contrast**	0.53 (0.06, 0.88)	0.83 (0.70, 0.90)	0.09 (0.07, 0.11)	0.18 (0.14, 0.24)	0.09 (0.02, 0.22)	0.66 (0.55, 0.83)	0.07 (-0.03, 0.17)	-48.48 (-56.42, -42.32)	94.10 (73.37, 129.44)	1.00 (1.00, 1.00)
**Strength**	0.29 (0.02, 0.71)	0.30 (0.05, 0.56)	10.93 (3.06, 199.52)	4.33 (0.97, 95.72)	2.45 (0.43, 52.27)	1.97 (1.62, 2.43)	-0.01 (-0.30, 0.28)	-86.04 (-91.16, -80.28)	616.59 (407.18, 1031.44)	1.00 (1.00, 1.01)

shape

Name	ICC inter (95% CI)	ICC intra (95% CI)	wlCV (95% CI)	blCV (95% CI)	bCV (95% CI)	RC (95% CI)	bias (95% CI)	-LoA (%) (95% CI)	+LoA (%) (95% CI)	R-hat (range)
**Elongation**	0.39 (0.02, 0.85)	0.84 (0.73, 0.91)	0.69 (0.40, 12.37)	0.69 (0.50, 1.40)	0.22 (0.04, 0.71)	0.29 (0.24, 0.36)	0.02 (-0.02, 0.06)	-25.09 (-30.27, -21.15)	33.49 (26.83, 43.42)	1.00 (1.00, 1.00)
**Flatness**	0.31 (0.02, 0.84)	0.84 (0.73, 0.91)	0.19 (0.15, 0.24)	0.36 (0.29, 0.49)	0.10 (0.02, 0.36)	0.34 (0.28, 0.42)	-0.01 (-0.06, 0.04)	-28.87 (-34.57, -24.39)	40.59 (32.26, 52.84)	1.00 (1.00, 1.02)
**LeastAxisLength**	0.48 (0.02, 0.92)	0.91 (0.84, 0.95)	0.04 (0.04, 0.05)	0.13 (0.11, 0.17)	0.04 (0.01, 0.14)	0.31 (0.26, 0.39)	-0.02 (-0.07, 0.02)	-26.44 (-32.00, -22.52)	35.94 (29.06, 47.06)	1.01 (1.00, 1.02)
**MajorAxisLength**	0.65 (0.04, 0.95)	0.96 (0.92, 0.98)	0.02 (0.02, 0.03)	0.10 (0.09, 0.13)	0.03 (0.00, 0.10)	0.21 (0.18, 0.27)	-0.01 (-0.05, 0.02)	-19.29 (-23.44, -16.32)	23.90 (19.51, 30.61)	1.00 (1.00, 1.02)
**Maximum2DDiameterColumn**	0.50 (0.02, 0.91)	0.93 (0.88, 0.96)	0.03 (0.02, 0.04)	0.11 (0.09, 0.14)	0.03 (0.00, 0.10)	0.28 (0.23, 0.35)	-0.02 (-0.06, 0.03)	-24.64 (-29.67, -20.66)	32.69 (26.05, 42.19)	1.00 (1.00, 1.01)
**Maximum2DDiameterRow**	0.46 (0.03, 0.92)	0.94 (0.89, 0.97)	0.03 (0.02, 0.03)	0.11 (0.09, 0.14)	0.03 (0.01, 0.09)	0.26 (0.22, 0.33)	0.02 (-0.02, 0.05)	-23.12 (-27.80, -19.57)	30.07 (24.32, 38.51)	1.00 (1.00, 1.01)
**Maximum2DDiameterSlice**	0.60 (0.05, 0.94)	0.95 (0.90, 0.97)	0.02 (0.02, 0.03)	0.09 (0.08, 0.12)	0.03 (0.01, 0.09)	0.21 (0.18, 0.27)	-0.01 (-0.04, 0.02)	-19.31 (-23.36, -16.29)	23.94 (19.47, 30.48)	1.00 (1.00, 1.01)
**Maximum3DDiameter**	0.68 (0.06, 0.96)	0.96 (0.93, 0.98)	0.02 (0.02, 0.03)	0.10 (0.08, 0.13)	0.03 (0.01, 0.10)	0.21 (0.17, 0.26)	-0.01 (-0.04, 0.02)	-18.79 (-22.80, -15.86)	23.13 (18.85, 29.54)	1.00 (1.00, 1.01)
**MeshVolume**	0.70 (0.09, 0.96)	0.96 (0.93, 0.98)	0.02 (0.02, 0.03)	0.10 (0.08, 0.13)	0.03 (0.01, 0.10)	0.50 (0.41, 0.63)	-0.00 (-0.08, 0.07)	-39.55 (-46.74, -33.89)	65.42 (51.27, 87.77)	1.00 (1.00, 1.01)
**MinorAxisLength**	0.56 (0.02, 0.93)	0.94 (0.90, 0.97)	0.03 (0.02, 0.03)	0.10 (0.08, 0.13)	0.03 (0.00, 0.09)	0.22 (0.18, 0.27)	0.01 (-0.02, 0.04)	-19.74 (-23.96, -16.65)	24.59 (19.97, 31.50)	1.00 (1.00, 1.02)
**Sphericity**	0.21 (0.01, 0.79)	0.86 (0.76, 0.92)	0.15 (0.12, 0.19)	0.31 (0.25, 0.41)	0.07 (0.01, 0.24)	0.12 (0.10, 0.15)	0.00 (-0.02, 0.02)	-11.33 (-13.95, -9.45)	12.77 (10.44, 16.21)	1.00 (1.00, 1.01)
**SurfaceArea**	0.68 (0.08, 0.96)	0.96 (0.93, 0.98)	0.02 (0.01, 0.02)	0.08 (0.07, 0.11)	0.02 (0.00, 0.08)	0.35 (0.29, 0.44)	-0.00 (-0.06, 0.05)	-29.63 (-35.62, -25.22)	42.11 (33.72, 55.32)	1.00 (1.00, 1.01)
**SurfaceVolumeRatio**	0.55 (0.05, 0.93)	0.93 (0.87, 0.96)	0.09 (0.07, 0.11)	0.28 (0.22, 0.37)	0.09 (0.02, 0.29)	0.21 (0.17, 0.26)	-0.00 (-0.03, 0.03)	-18.98 (-22.95, -15.95)	23.43 (18.98, 29.79)	1.00 (1.00, 1.01)
**VoxelVolume**	0.68 (0.06, 0.96)	0.96 (0.93, 0.98)	0.02 (0.02, 0.03)	0.10 (0.08, 0.12)	0.03 (0.01, 0.10)	0.49 (0.40, 0.61)	-0.00 (-0.08, 0.07)	-39.03 (-45.86, -33.22)	64.01 (49.75, 84.69)	1.00 (1.00, 1.01)
