# Supplementary material for: Inter- and Intra-Patient Repeatability of Radiomic Features from Multiparametric Whole-Body MRI in Patients with Metastatic Prostate Cancer
Source: Cancers (Basel). 2024 Apr 25;16(9):1647. doi: 10.3390/cancers16091647 (PMC11083580; doi:10.3390/cancers16091647)
Supplement: Supplementary file 1 [file cancers-16-01647-s001.zip › Supplementary S6.pdf]

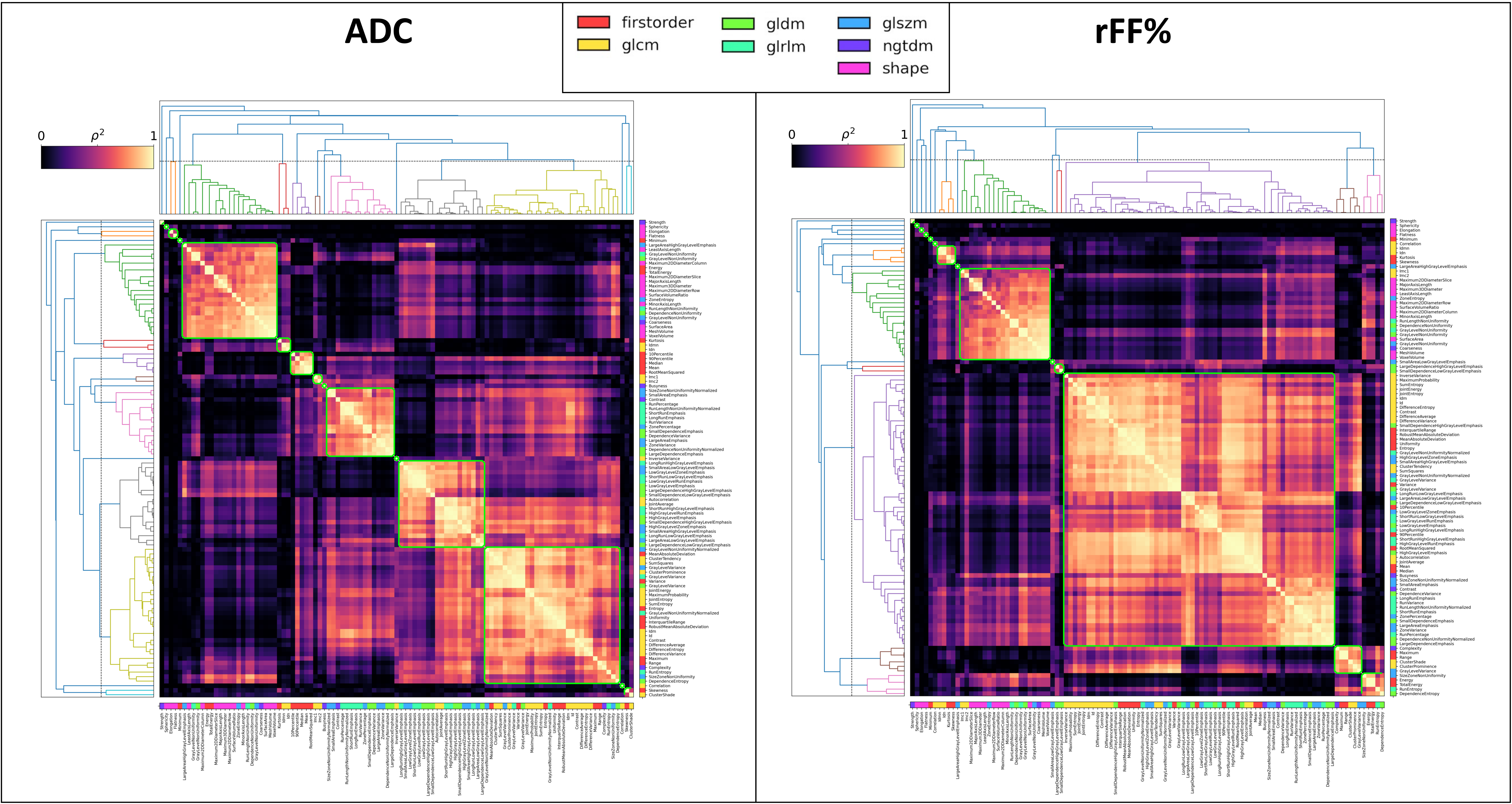
ADC
firstorder
glcm
gldm
glrlm
glszm
ngtdm
shape
rFF%
0
ρ²
1
0
ρ²
1
