# Supplementary figures and images for: Inter- and Intra-Patient Repeatability of Radiomic Features from Multiparametric Whole-Body MRI in Patients with Metastatic Prostate Cancer
Source: Cancers (Basel). 2024 Apr 25;16(9):1647. doi: 10.3390/cancers16091647 (PMC11083580; doi:10.3390/cancers16091647)

ADC

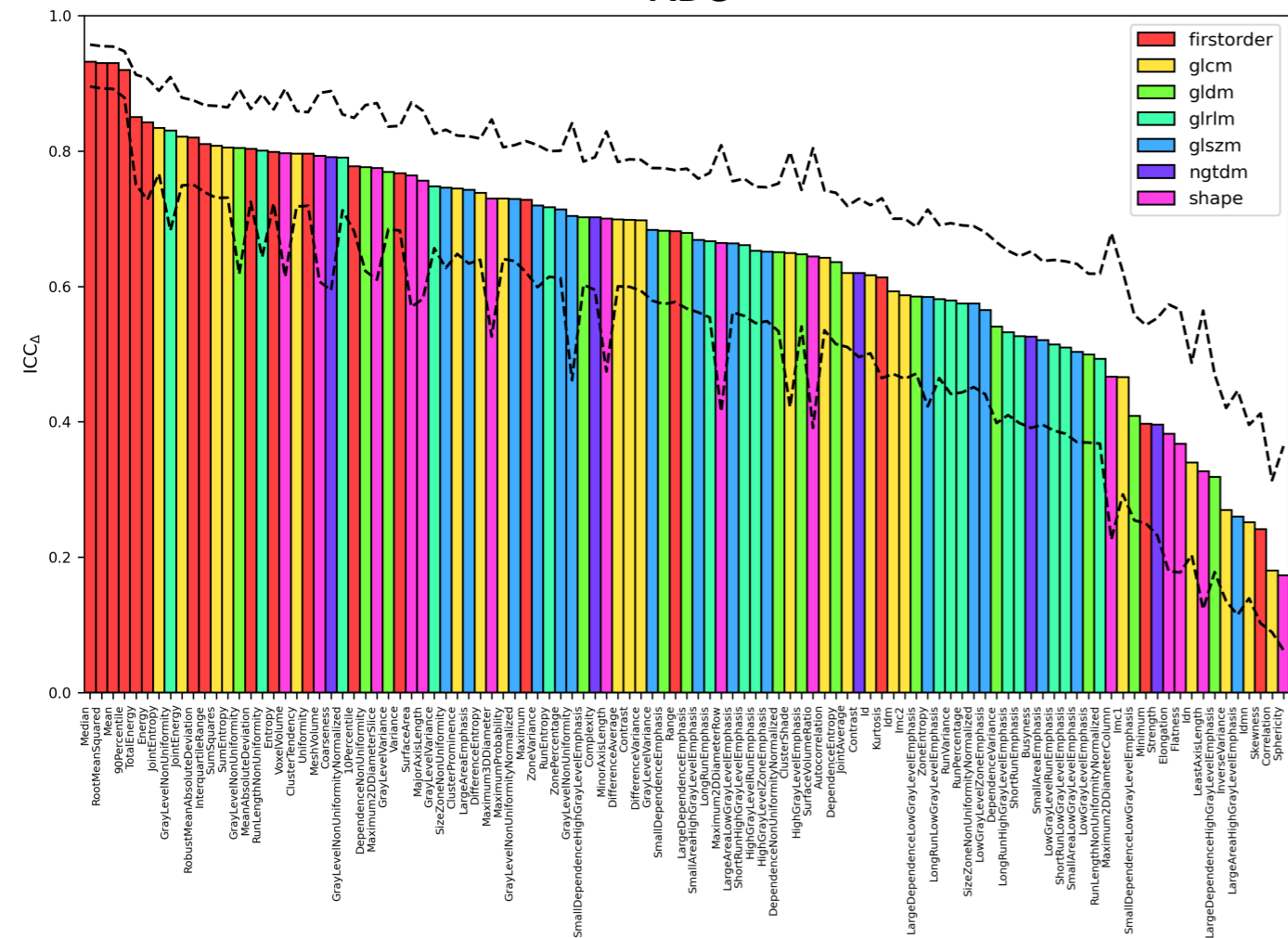

rFF%

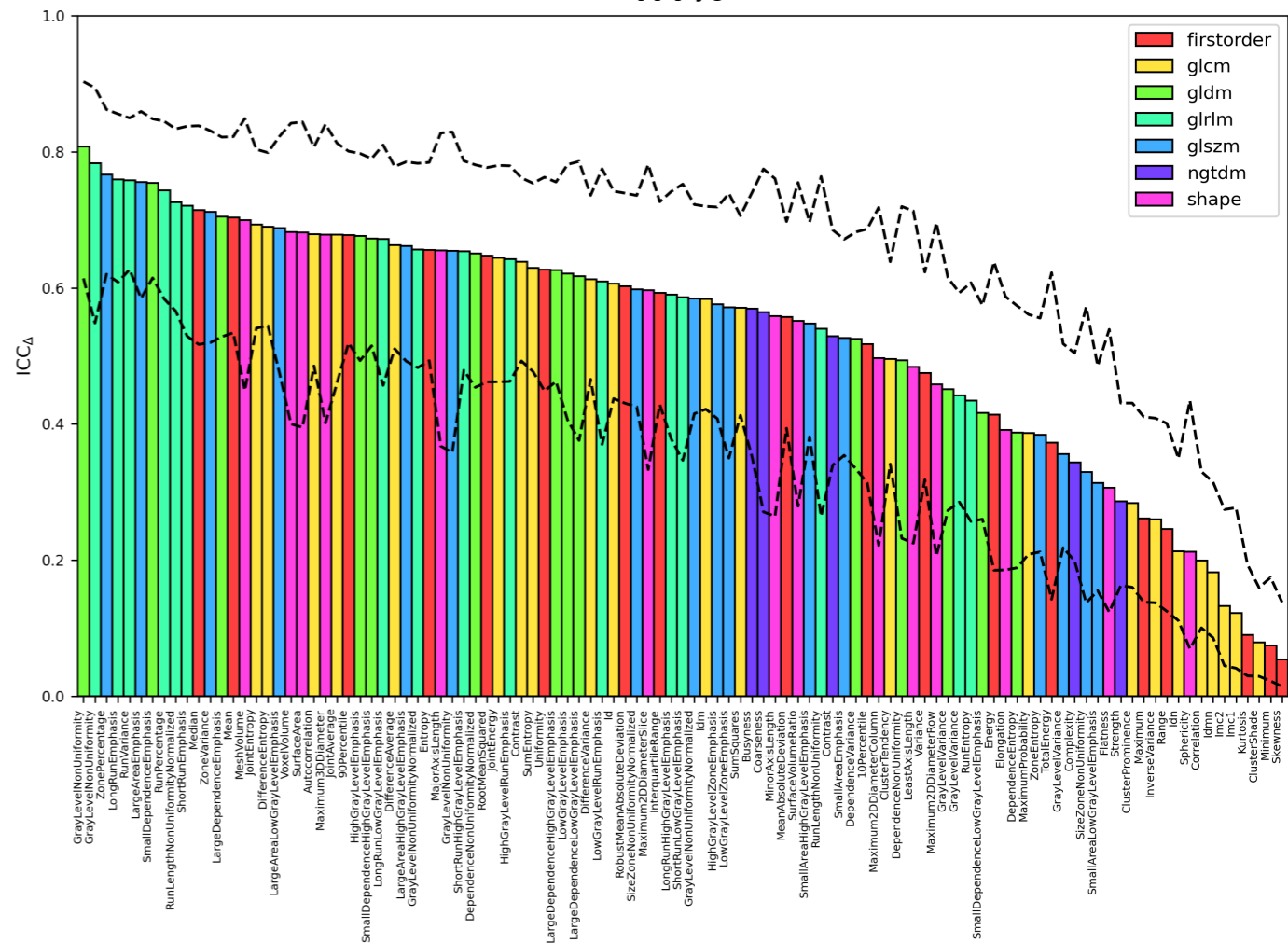

ADC

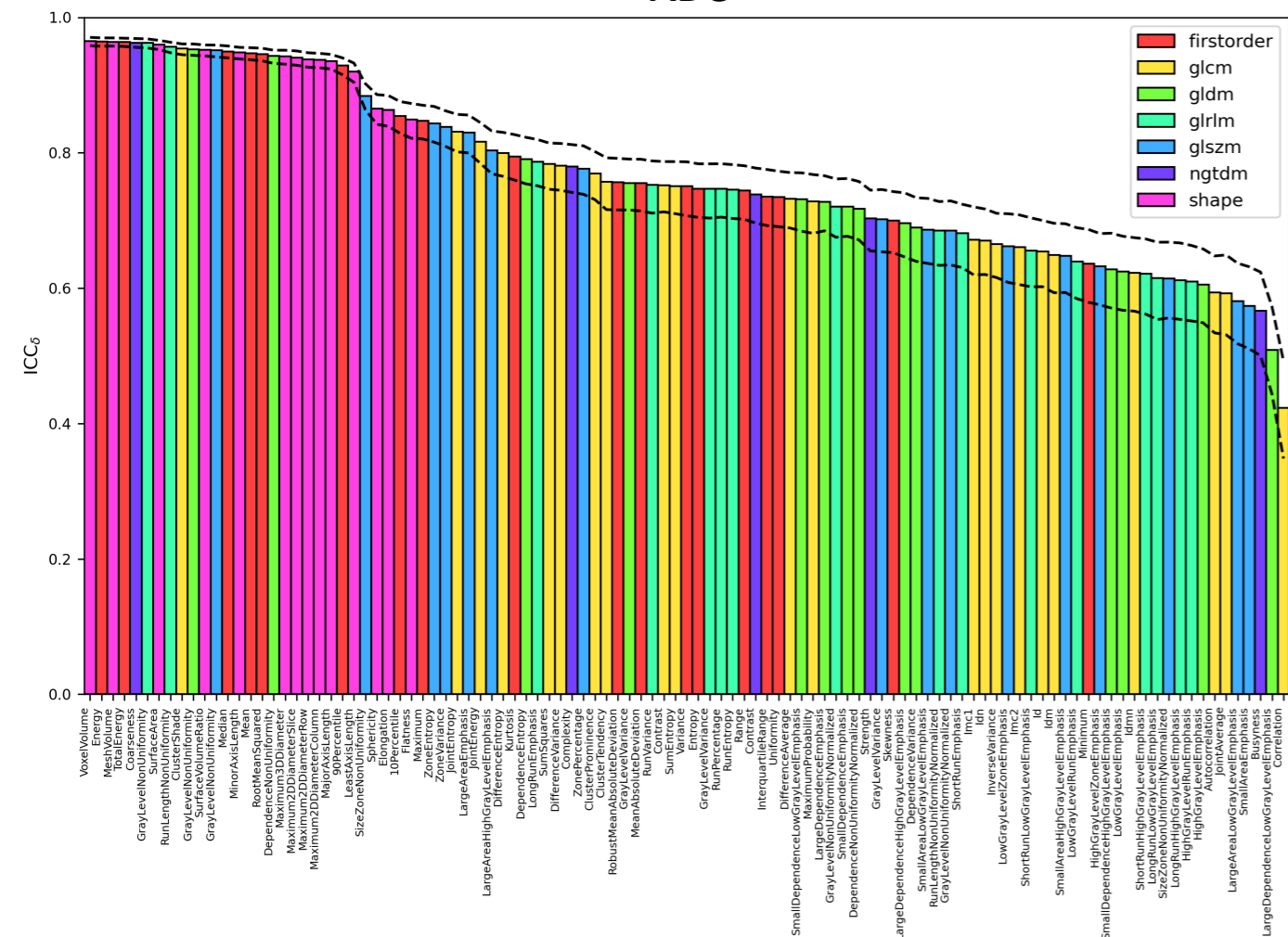

rFF%

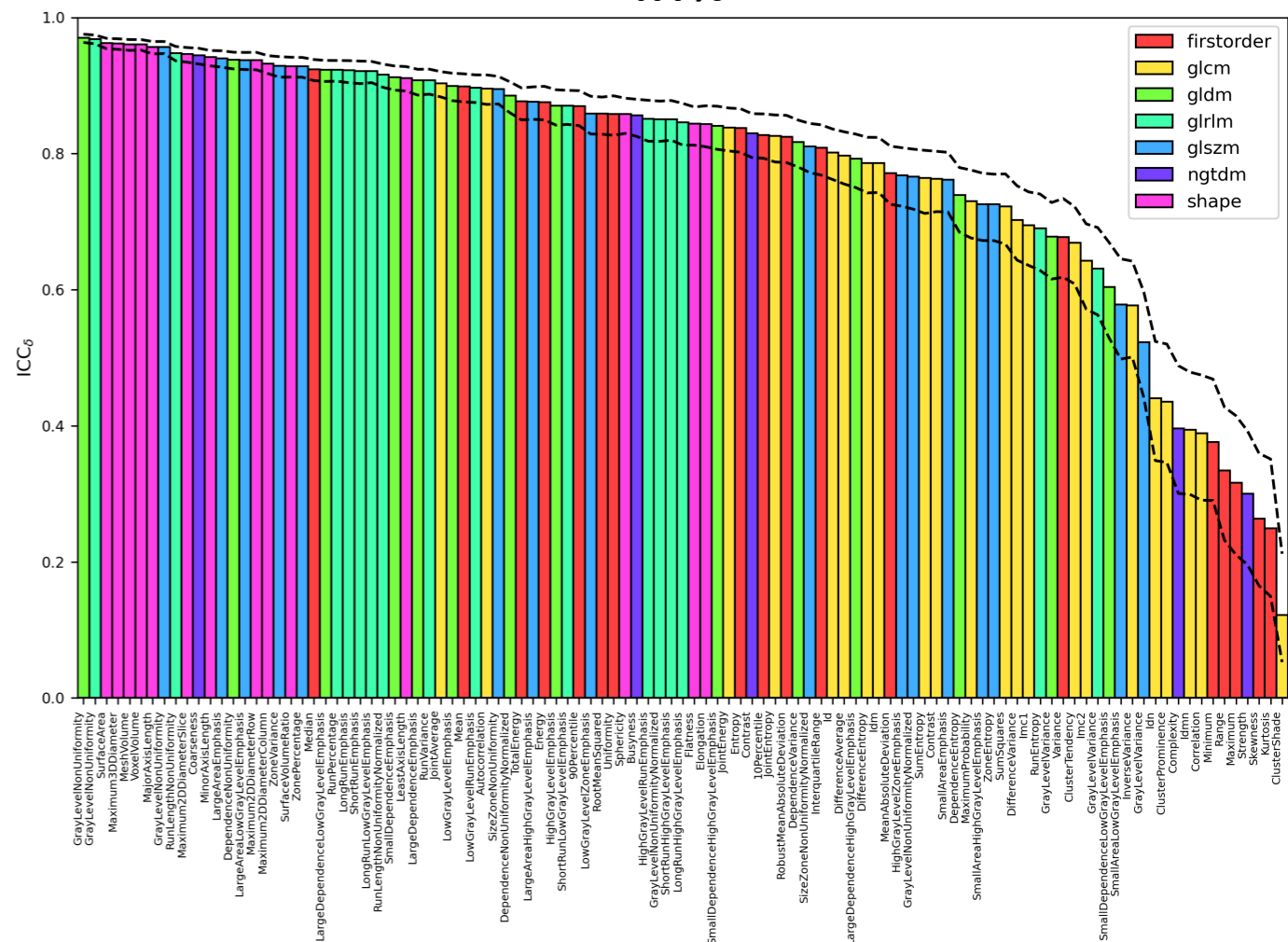

Supplement: Supplementary file 1 [file cancers-16-01647-s001.zip › Supplementary S7.pdf]
